# Transforming retraining: Supporting racehorses to have successful second careers

**DOI:** 10.1371/journal.pone.0355509

**Published:** 2026-08-19

**Authors:** Jane M. Williams, Saranna Jordan, Laura Friend, Emily Kay, Matilda Edmunds, Kathryn Nankervis, Jessica Cheung, Gillian Tabor

**Affiliations:** 1 Equine Department, Hartpury University, Gloucester, United Kingdom; 2 Hong Kong Jockey Club, Hong Kong, China; Universidade do Porto Instituto de Biologia Molecular e Celular, PORTUGAL

## Abstract

Optimizing long-term welfare outcomes for Thoroughbred racehorses following retirement from racing is a priority across the global horse racing industry. Following their racing career, horses are required to adapt to a markedly different lifestyle, involving substantial physical, behavioural, and mental adjustment. This study consulted experienced retrainers to identify factors that inform successful retraining and transition of former racehorses to promote positive lifelong outcomes beyond racing. An online survey of 100 global stakeholders experienced in retraining former racehorses explored common challenges, practices, and perceptions associated with transition. The survey results informed a subsequent Delphi consultation involving 27 experienced racehorse retrainers and musculoskeletal therapists, to establish consensus on which factors were essential to underpin successful transition. Survey data were analysed using frequency analysis and inductive content analysis, while the Delphi consultation used content validity ratios to determine consensus across behavioural, physical, exercise, and management domains relevant to retraining. Survey respondents identified four key themes influencing successful retraining outcomes: the time allocated for retraining, the people involved in the process, the horse’s prior racing and management history, and the overall approach taken to retraining. The Delphi consultation achieved consensus on seven areas considered essential to successful transition: feeding regime, rider skill, handler expertise, type of exercises undertaken, assessment and modification of the horse’s shape, assessment and modification of posture, and provision of turnout. These findings emphasize the importance of addressing both physical and mental adaptation during transition. The versatility of Thoroughbreds was evident, with retrainers commonly preparing horses for eventing, dressage, and leisure riding homes. Successful transitions closely aligned individual horse capabilities with future owner expectations. Participants highlighted the need for increased owner education, improved access to professional expertise, and greater financial support for retrainers. Implementing evidence-informed, multidisciplinary transition pathways may enhance retraining practices and promote sustainable, positive outcomes for former racehorses beyond racing.

## Introduction

Following retirement from their racing careers, racehorses are required to adapt to a new lifestyle. This process, hereafter defined as transition, requires considerable physical and mental adaptation as horses adjust to the demands of different exercise and management expectations. Managing these adaptations successfully presents challenges for those caring for horses during retraining throughout the transition period [1]. In the UK alone, approximately 7000 horses leave racing each year; the age horses leave racing varies based on performance, soundness and post-racing opportunities but generally flat racehorses retire or go to stud between 3 and 5 years of age, while National Hunt (jump) racehorses retire later between 8 and 10 years of age [2,3]. Around 40% enter retraining for second careers, highlighting the importance of effective transition to safeguard the health and welfare of the Thoroughbreds involved [4,5]. Consequently, optimizing long-term outcomes for former racehorses has been identified as a priority within horse racing’s social licence to operate by industry stakeholders, researchers, and the public [6–9].

Supporting racehorse aftercare as part of racing’s lifetime commitment to Thoroughbred welfare is a strategic priority in British racing and across global racing jurisdictions [10]. This commitment is articulated in the British Horseracing Authority’s Horse Welfare Board *A Life Well Lived Strategy* [9] and reinforced within the 2024–2026 strategy of the British racehorse aftercare charity Retraining of Racehorses [4,10,11]. Internationally, racing jurisdictions have implemented comparable programmes, such as the Hong Kong Jockey Club’s Retired Racehorse Retraining Programme, while organizations including the International Forum for the Aftercare of Racehorses provide guidance and promote best practice across the sector [8,12,13]. Despite widespread agreement that effective early retraining is essential to maximize horses’ potential for successful second careers and positive lifelong welfare outcomes [6,10], research investigating racehorse aftercare remains limited. In particular, there is a lack of evidence describing how retraining programmes are designed, which features they incorporate, and how they are implemented in practice [6,14].

An improved understanding of career, health, and welfare outcomes for former racehorses could be achieved by identifying common challenges and features of successful retraining [1,15]. Successful retraining is likely dependent on recognising horses’ physical, behavioural, and training needs throughout the transition process, although this has yet to be systematically confirmed by retrainers. While centres with substantial expertise exist, most former racehorses are sourced directly from trainers or private sellers [16], as a result horses often transition under the care of less experienced owners and handlers. Effective knowledge exchange with experienced retraining organizations may therefore enhance welfare outcomes.

A fundamental aspect of transition is the change in exercise demands between racing and potential second careers [17–20]. Racing prioritises galloping performance, whereas many second careers, such as dressage and eventing, require collection, postural change, and different musculoskeletal loading. Training programmes should therefore consider individual horse’s conformation, history, and adaptive capacity to support career longevity. Although Thoroughbreds are versatile [5,6], not all horses will be suited to all outcomes, reinforcing the importance of evidence-informed decision-making during transition [4,7,10].

Understanding how different professionals contribute to successful transition is warranted. Retraining teams may include veterinarians and allied professionals such as farriers and musculoskeletal therapists [10,18], however their role within transition remains poorly defined. This study consulted professionals involved in retraining former racehorses to identify key features of transition, understand common physical and behavioural challenges within the process, and strategies used to overcome these, to determine what an optimal transition involves.

## Materials and methods

A two-stage, mixed-methods study design investigated factors underpinning successful transition of former racehorses into second careers. Stage 1 comprised an international online survey of stakeholders involved in racehorse retraining, using an inductive approach to identify key themes relevant to the transition process. Findings from Stage 1 informed Stage 2, a modified, iterative Delphi consultation designed to achieve expert consensus on the essential components of transition across key professional groups, including retrainers, veterinarians, and physiotherapists. The Delphi method was selected due to limited empirical evidence and variation in professional opinion regarding optimal racehorse transition practices. The Delphi incorporated integrated discussion groups with UK-based professionals actively engaged in racehorse retraining and followed a multi-modal approach previously used to develop a tool for monitoring osteoarthritis pain in horses [20]. Collectively, the two stages aimed to establish agreement on the timelines, activities, and professional inputs required to support effective transition of racehorses into the next stage of their lives. The Phase 1 survey took place from April-June 2023. The Delphi Round 1 was conducted in October 2023, with subsequent focus groups occurring approximately 8 weeks later, and Round 2 completed 8 weeks following these.

The study was approved by the Hartpury University Ethics Committee (ETHICS2022−14). Written information was provided to all participants outlining the rationale and aims of the study and subsequent informed consent was obtained prior to participation.

### Stage 1: Global survey

#### Survey design and distribution.

A link to an online questionnaire (Qualtrics XM™, Seattle, WA, USA) (S1) containing 30 questions including 2 closed questions, 8 multiple choice questions, 4 check box questions, 6 Likert scale questions, and 12 open free text questions was distributed to voluntary participants recruited via relevant equestrian, racing and racehorse retraining social media sites. To be eligible to participate, respondents were required to be over 18 years of age and be actively involved in retraining or supporting transition for racehorses into their second careers. The draft survey was tested by five experienced equestrian researchers and edited to correct any errors before being fully deployed. The survey was launched on 26^th^ May 2023 and was live for 42 days.

The questionnaire was split into five major sections:

1. Respondent demographics: Respondents provided their qualifications, experience, and geographical location of staff involved in the retraining process,
2. Factors that can influence transition: Respondents were provided with lists of key physical attributes, factors that could affect behaviour and health and injury related factors encountered in race training that could influence the retraining process and success in horses’ future careers health. They were asked which they felt could influence the retraining process most, scored: always likely, very likely, likely, sometimes likely to never likely to be influential to successful transition; respondents could also select ‘unsure’ as an alternative option for each attribute.
3. Transition structure: Respondents were asked when they would start the transition process, which activities they would include and the duration of transition on average using the same Likert scale approach.
4. Physical and behavioural indicators of progress within transition: Respondents were provided with a range of physical and behavioural attributes they could encounter in Thoroughbreds during transition and were asked to confirm if they used these to evaluate horses’ progress. They were also asked if they used any equipment or technology to record or monitor horses’ progress or to measure the success of transition.
5. Decision-making for future careers: Respondents were asked to rate how likely they would be to use a selection of factors when selecting Thoroughbreds to retrain and when selecting suitable future careers for individual horses.

#### Survey data analysis.

Data were exported from Qualtrics to Microsoft Excel™ Version 2010 (Redmond, WA, USA). Frequency analysis identified the demographic characteristics of respondents and the regularity with which different behavioural, physical, exercise and training, and assessment measures were likely to be used by respondents within the transition process. For Likert scale questions, the most common approaches used by respondents across retraining, were assessed through the calculation of the percentage response for each variable after removal of any ‘unsure’ responses; the Likert scale responses were then assigned numerical ratings to enable median and interquartile ranges to be calculated. Open questions within the survey were evaluated through inductive conventional content analysis using a grounded theory approach to identify themes arising from the data [21].

### Stage 2: Delphi consultation

An iterative Delphi methodology was employed to achieve expert consensus on best practice for the early retraining and transition of former racehorses. Delphi studies outline the opinions of the people or ‘experts’ selected to take part in them and are commonly employed where knowledge is incomplete or different opinions may exist to reach a consensus on which clinical guidelines or performance indicators should be used to address an identified challenge [4,22–26]. The Delphi approach was selected due to limited empirical evidence and variation in professional opinion regarding optimal transition practices [22,23]. Findings from Stage 1 informed the development of the Delphi surveys, which explored challenges, timelines, and activities associated with successful transition. The Delphi comprised two quantitative survey rounds, with an intermediate qualitative focus group stage used to refine and contextualize survey domains. This approach aligns with established Delphi methodology for achieving consensus where knowledge is incomplete or practice varies [21–31].

The Delphi process was designed and reported in alignment with the Guidance on Conducting and REporting DElphi Studies (CREDES) (S2) [28]. Expert opinion was gathered through anonymous online surveys, with controlled feedback provided between rounds to allow participants to review group-level responses and revise their ratings accordingly [23,29]. Quantitative ratings remained anonymous throughout all rounds to minimize dominance effects, while structured online discussion groups were facilitated to support clarification and refinement of domains without hierarchical influence.

#### Participant selection.

Participants were recruited via convenience sampling from UK-based professionals actively engaged in teams responsible for former racehorse retraining (S3). Three core professional groups were targeted to provide a multidisciplinary perspective: retrainers, veterinary surgeons, and equine physiotherapists. Eligibility criteria required participants to have been directly involved in the transition and retraining of a minimum of 10 former racehorses, including contributing to decision-making related to management, training, or rehabilitation, and to be over 18 years of age. Equine physiotherapists also had to hold a relevant qualification eligible for entry on the Register of Animal Musculoskeletal Physiotherapists. Veterinary surgeons were also required to be a member of the British Equine Veterinary Association. Informed consent was obtained prior to participation.

A minimum Delphi panel size of between 10–15 experts with an expected attrition rate of up to 20% was deemed acceptable due to the specialized nature of the topic being evaluated [32,33]. Delphi participants were recruited through convenience and opportunistic sampling via professional networks and relevant social media platforms, including the Association of Chartered Physiotherapists in Animal Therapy (ACPAT). Although veterinary surgeons were targeted for recruitment, no eligible veterinarians responded to the invitation. The final Delphi panel therefore comprised physiotherapists and retrainers only.

#### Delphi round 1: survey and analysis.

In Round 1, participants completed an online survey rating the importance of behavioural, physical, exercise, management, and health-related factors influencing successful transition. Participants rated each domain as: (1) essential, (2) not essential but important, or (3) not essential. Content Validity Ratios (CVR) were calculated as a complementary measure to confirm that agreement exceeded chance levels [34,35], Participants were also asked to indicate when specific exercise activities should be introduced, how progress was monitored, indicators of successful retraining, factors prompting deviation from a standard retraining plan, and perceived challenges during transition. Open-ended responses allowed participants to suggest additional factors not captured within predefined domains.

The CVR was calculated as:


$$\begin{array}{c} CVR=\frac{ne-\frac{N}{2}}{(\frac{N}{2})} \end{array}$$

Where “CVR”: Content validity ratio, “ne” is the number of essential members and “N” is the number of panel members [35,36].

#### Intermediate qualitative focus group discussions.

Intermediate focus groups were planned a priori to explore the reasoning underpinning expert opinions and to synthesise nuanced perspectives, informing refinement of the Round 2 survey. Two online focus groups were conducted and attended by ten UK-based Association of Chartered Physiotherapists in Animal Therapy (ACPAT) Chartered Physiotherapists (five in each group), recruited via professional networks. Sessions were held via Microsoft Teams™ (Redmond, WA, USA) and facilitated by the lead researcher (GT). Discussions began with open-ended questions exploring participants’ experience of racehorse transition and expanded to examine key domains identified in Round 1. Focus group sessions were audio-recorded and transcribed verbatim. Data were analysed thematically using a grounded theory approach, progressing from open to focused coding in alignment with Clarke and Braun’s [36] six-phase framework. Transcripts were analysed and triangulated by GT and JMW. Both researchers adopt a pragmatic epistemological stance, focusing on generating findings that are useful in applied settings. Their shared perspective supports horses being ridden, provided that management and training approaches prioritise a good quality of life and promote positive welfare outcomes.; This thematic analysis served to contextualise quantitative findings from Round 1, identify emerging domains, and refine item wording for the Round 2 survey.

#### Delphi round 2: Survey and final consensus.

Following Round 1, fourteen domains met the threshold for inclusion (≥70% of participants rated as essential, scored a CVR ≥ 0.70), with two additional domains proposed by participants. These were reviewed alongside focus group findings and presented to the full Delphi panel in Round 2 for re-rating. Consensus was defined *a priori* using agreement by ≥70% of panel members that an area was agreed to be essential within retraining, a threshold commonly adopted in Delphi studies [30,31] where consensus can vary between 56 and 90% [32,37,38]. Participants rated if each domain was essential using a 9-point Likert scale: 0- not essential to 9: always essential to allow for more nuanced opinions [37,38]. Answers scoring 7, 8, or 9 on the scale were deemed to exceed the threshold for an essential rating to facilitate CVR calculation as outlined in Stage One [33]. Content Validity Ratios (CVR) were calculated as a complementary measure to confirm that agreement exceeded chance levels [34,35], A critical value of 0.7 was selected as this represented the suggested CVR threshold of 0.68 for a retained panel size of 12 members as recommended by Lawshe [39] and as this aligned to stablished critical values based on panel size adopted in other Delphi studies [34,35,40–42]. Items meeting both the percentage agreement threshold and the minimum CVR value were retained for subsequent rounds or final inclusion.. Final inclusion of domains was determined using both quantitative consensus ratings and qualitative comments, in line with recommended Delphi reporting standards [34,41]. Panel size and retention across rounds are reported in the Delphi Results section.

## Results

### Survey results

**Fig 1:**
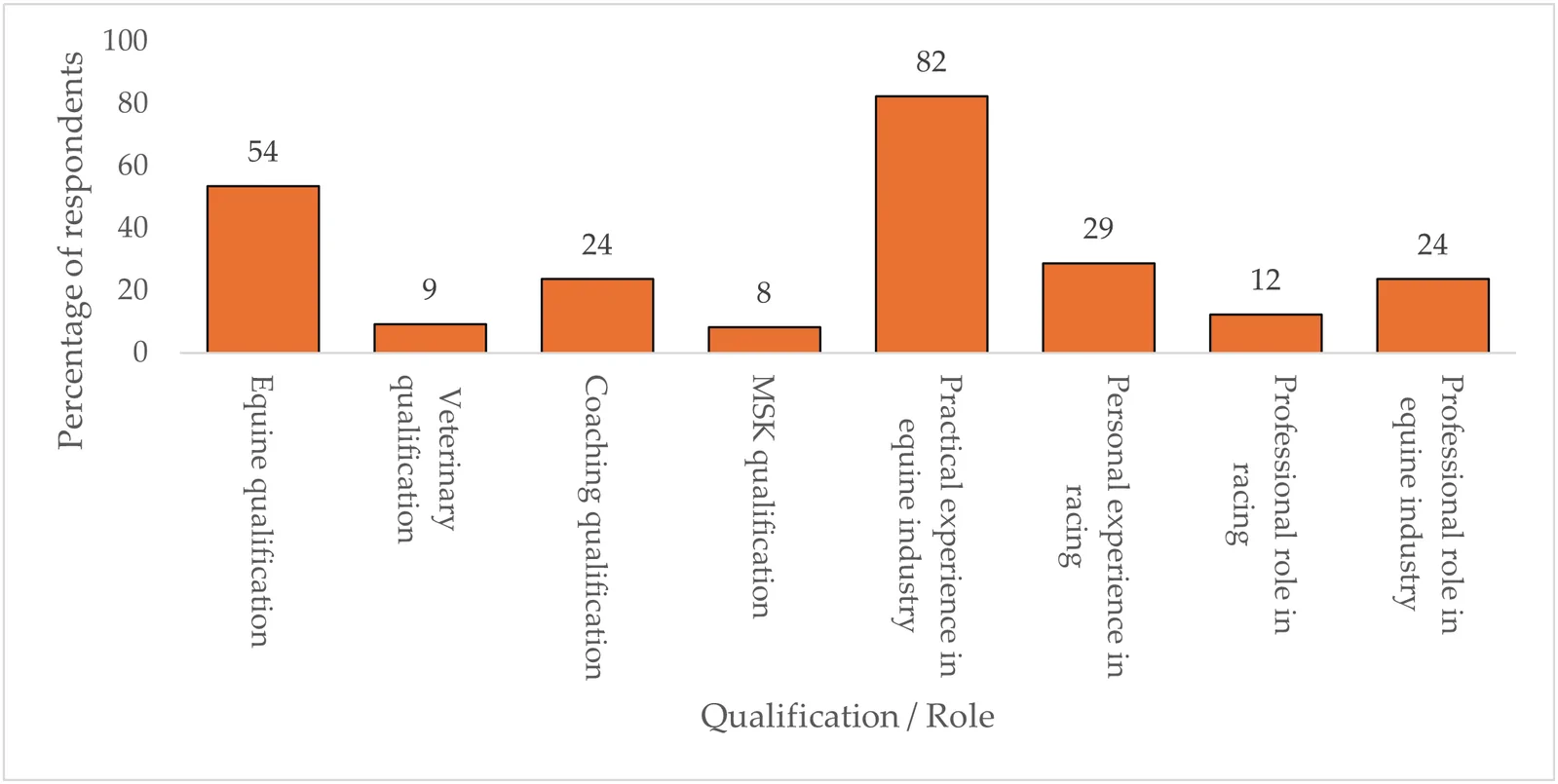
Respondents’ qualifications and roles. Survey respondents (n = 100) were asked to select all qualifications and equestrian roles that applied to them. Note: Multiple options could be selected. MSK: musculoskeletal therapist.

A total of 110 people responded to the survey; of these, 100 complete responses were taken forward for analysis; 70% were based in the UK, 20% in Australia / New Zealand, with 5% located in North America and 5% in China. Most respondents had practical or personal experience in either racing or the broader equine industry (Fig 1); 62% had experienced transition with less than 5 horses, 18% of the remaining respondents had retrained between 6 and 10 horses, and the remaining 20% had retrained more than 10 horses.

#### Retraining history.

**Fig 2:**
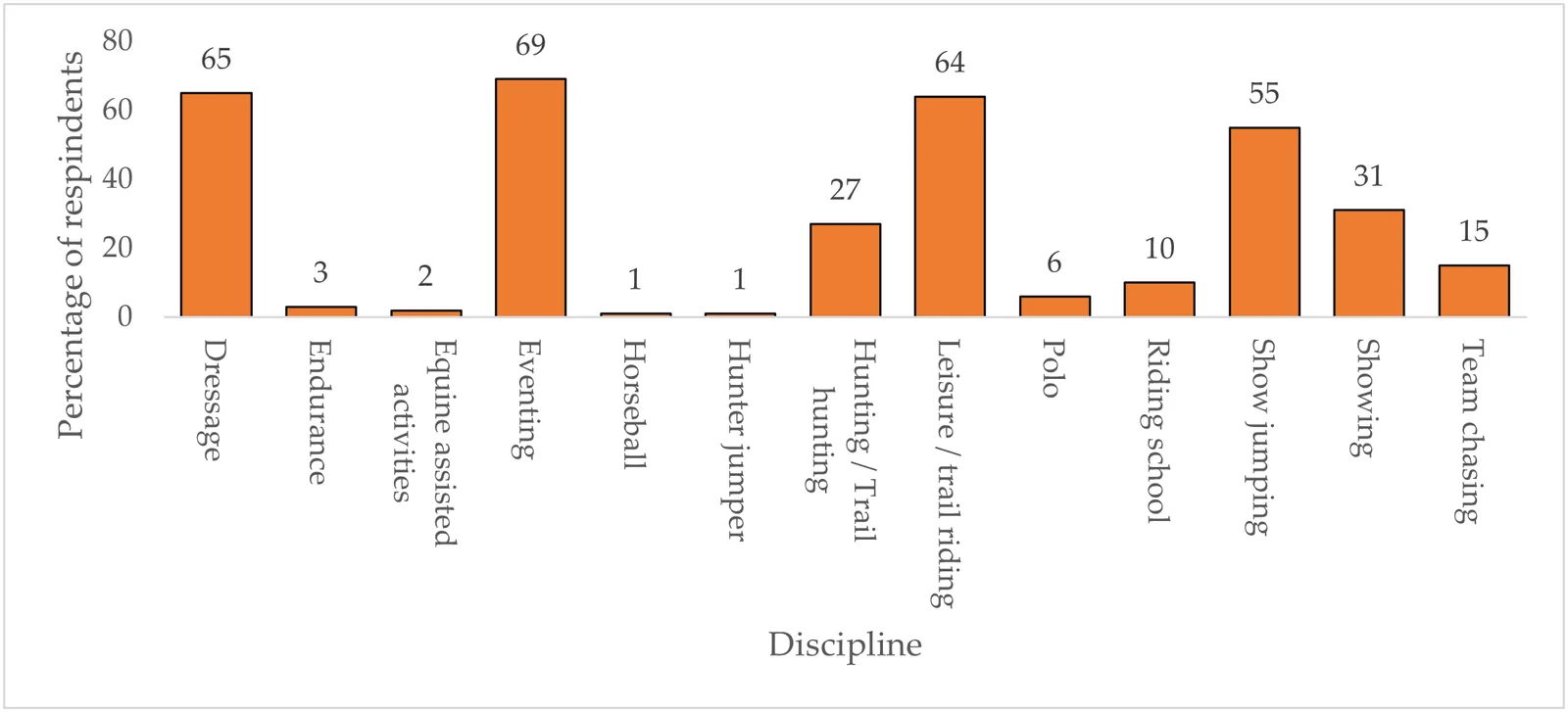
Disciplines retrainers self-reported they targeted as suitable career destinations for former racehorses. Survey respondents (n = 100) were asked to identify which disciplines they would most frequently tailor retraining of former racehorses for, to prepare them for a potential successful second career. Respondents were also provided with the opportunity to identify additional disciplines or activities which were not listed within an ‘other’ category. The other activities included western riding and polocrosse. Showing: Horse shows that can be ridden or in hand where horse conformation, performance and capacity are judged; Team chasing: Cross-country jumping competition that takes place in teams of four often over natural obstacles and terrain; Endurance: competitive long distance riding events ranging from 16 to 160 km in one day.

Most respondents (66%) had retrained racehorses for over 5 years and did this working on a one-to-one basis with individual horses. A further 20% worked in a private training yard (10%) or retrained Thoroughbreds professionally working with multiple horses (10%). Less (3%) worked directly with a racehorse trainer or worked on a specific Thoroughbred retraining yard (2%); with the remaining 8% either retraining racehorses for their own personal use or within a competition yard retraining racehorses for other disciplines. The five most common reasons for being involved in retraining racehorses were to provide the horses with a good quality of life (67%), to produce or to have their own horse (60%), individual attachment to a specific racehorse (34%), competitive opportunities (32%) or because they were approached to retrain the horse (29%). Few respondents were involved with retraining as their core business (9%) or via their employment (8%); 20% were involved due to specific competitive opportunities that were targeted for former racehorses. A further 16% listed other reasons for their involvement, including their love of the thoroughbred and the breed’s versatility and affordability. Former racehorses were retrained with the goal to be able to enter a variety of equestrian disciplines and activities (Fig 2).

#### Factors influencing retraining.

A horse’s temperament (79%), physicality (77%) and behaviour (76%) (S4 Table S1) were considered influential by retrainers when selecting Thoroughbreds that could transition successfully. Individual horses’ trainability (91%), physical health (91%) and mental wellbeing mostly that influenced how long respondents kept a former racehorse for retraining (S5 Table S2). Time spent in the paddock or at free exercise (86%), different types of work (85%) and changes in the horse’s shape (79%) were highlighted as key physical attributes that influenced retraining success (Table 1). Significant factors that respondents felt influenced former racehorse behaviour and transition were changes in time spent in the paddock or at free exercise (82%), a change in feeding concentrates (82%), and a change of daily routine from racing (78%) (Table 2). Respondents identified chronic lameness (86%), muscle pathology or disease (74%), and skeletal back pain (73%) as health-related factors encountered in race training which could influence the retraining process and success in former racehorses’ future careers (Table 3).

**Table 1:** Respondent views on the influence of management factors and horses’ physical attributes on the retraining process.

	Always (%)	Very likely (%)	Likely (%)	Unlikely (%)	Very unlikely (%)	Never (%)	Unsure (%)
Amount of exercise / training	36	41	19	4	0	0	0
Rider position due to the saddle	26	30	34	8	1	0	0
Different posture/ outline / frame	42	24	27	3	1	1	1
Shoeing/ trimming routine	29	32	28	9	0	1	0
Rider weight	27	23	32	17	0	0	1
Change in the horse’s shape	46	33	19	0	0	0	1
Training / exercise / yard routine	44	32	23	1	0	0	0
Time spent in paddock / free exercise	50	36	12	3	0	0	0
Time spent in the stable	35	29	25	9	0	1	1
Different types of work	44	41	14	1	0	0	0
Working on different surfaces	26	36	22	12	3	0	3
Bridle / bit used	22	18	46	12	1	0	1
Saddle used	29	31	28	9	3	0	0
Use of training aids	17	21	39	18	1	3	1

Survey respondents (n = 100) were asked to rate how likely they felt specific management factors and horses’ physical attributes that could be integrated into the transition process would be to a Thoroughbred’s future second career / rehoming success using a Likert scale (always to never including an option to state if they were unsure).

**Table 2:** Respondent views on the influence of management and behavioural characteristics on the retraining process.

	Always (%)	Very likely (%)	Likely (%)	Unlikely (%)	Very unlikely (%)	Never (%)	Unsure (%)
Change of feeding concentrates	42	40	14	3	0	1	0
Change of environment: climate	9	18	37	27	3	1	5
Change of environment: yard	24	35	32	6	0	0	3
Change of daily routine from racing	27	51	18	3	0	0	1
Change in transportation method	6	19	32	36	1	3	3
In/decreased changes in opportunities for contact with other horses	18	35	39	5	0	0	3
Change of feeding forage	40	27	28	5	0	0	0
Change of turnout / free exercise	58	24	15	1	0	0	1
Time spent in the stable	36	32	29	3	0	0	0
Change from working in a group to working alone	23	35	36	5	0	0	1
Frequency of exercise	26	37	31	5	1	0	0
Type of exercise	27	40	29	3	0	0	1
Rider / handler	41	26	26	5	0	0	3
Change of environment: other	29	31	36	4	0	0	0

Survey respondents (n = 100) were asked to rate how likely they felt the specific management factors could potentially influence horses’ behavioural characteristics during transition and if they felt they should be integrated into the transition process to expediate success using a Likert scale (always to never including an option to state if they were unsure).

**Table 3:** Respondent views on the influence of prior health and injury related factors on the retraining process.

	Always (%)	Very likely (%)	Likely (%)	Unlikely (%)	Very unlikely (%)	Never (%)	Unsure (%)
Muscle pathology / disease	28	46	17	8	0	0	1
Skeletal back pain	33	40	24	3	0	0	0
Pelvic/ LS / SI region pain	25	45	25	5	0	0	0
Limb: bone or joint issues / disease	28	38	26	6	0	0	1
Limb: tendon or ligament injury	25	30	21	18	3	0	4
Foot pain	24	37	28	10	0	0	0
Chronic lameness	60	26	10	4	0	0	0
Intermittent lameness	29	39	30	3	0	0	0
Poor performance	13	18	23	28	14	3	1
Breathing problems / airway disease	17	17	29	31	3	0	4
EIPH / Bleeder	13	12	32	35	5	0	4
Cardiovascular disease	23	25	32	13	1	0	5
Gastric ulcers	23	31	29	14	1	0	1
Muscular back pain	27	31	31	9	0	0	0

Survey respondents (n = 100) were asked to rate how likely they felt a horse’s prior health and injury history could influence the success of the transition process using a Likert scale (always to never including an option to state if they were unsure). LS: lumbosacral; SI: sacroiliac; EIPH: exercise induced pulmonary haemorrhage.

#### Transition structure.

**Fig 3:**
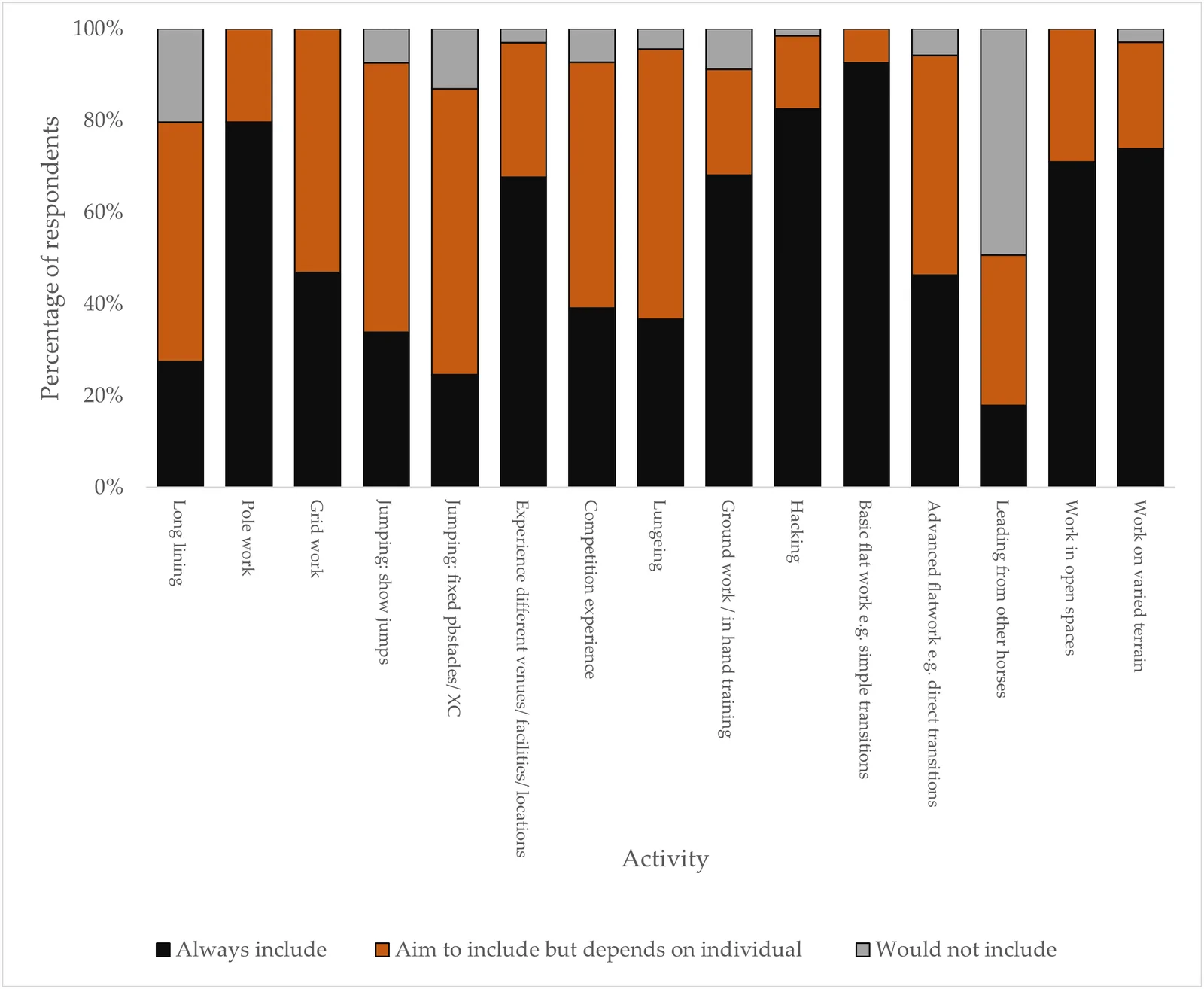
Frequency respondents reported including common equine training activities within transition. Respondent (n = 100) were asked which activities they would always include, may include depending on individual or, not include, within the transition process of former racehorses.

Approximately a quarter of respondents (29%) felt Thoroughbreds should start transition after a 6–8 week let down or spelling period (time out of work / turned out in a paddock) on their exit from racing. Less (17%) felt 4 weeks let down was sufficient, while 12% advocated 9–12 weeks out of work before starting retraining; a further 14% believed 4–6 months out of work was most appropriate, while a small number (1%) considered 13–16 weeks or >6 months out of work was required. In contrast, 25% started retraining immediately after a horse’s exit from racing. Respondents stated that they were always likely to include basic flatwork (93%), hacking (83%) and pole work (80%) in a retraining programme but were least likely to always include leading from another horse (18%), jumping fixed / cross-country obstacles (25%) or long lining (28%) within retraining (Fig 3). The average duration of transition varied across respondents with 7% stating it would be less than 3 months, 31% between 6–12 months, 13% between 4 and 6 months, 15% up to 2 years, and 27% recording transition would be longer than 2 years.

The timeframe respondents indicated they would most often introduce common activities into retraining programmes varied from immediately to more than two years; 10% of respondents stated they would base this on the individual horse, their stage of training, and their level of mental and physical maturity. Generally, groundwork was introduced in the first week, long lining after 1 week, pole work after 2 weeks, leading from other horses after 2 weeks, basic flatwork after 4 weeks, work on varied terrain after 4 weeks, and grid work after 8 weeks with an overall range for all these activities up to six months. Lungeing on average was reported to be introduced after 1 week, work in open spaces after 4 weeks, advanced flatwork after 6 weeks, and experience of different venues/places after 12 weeks, with timeframes for these activities ranging between the start of retraining and twelve months. Other activities including show jumping were introduced on average after 8 weeks, jumping cross-country obstacles after 12 weeks, and competition experience after 12 weeks), with ranges reported for these to be started after one month to up to three years from the start of retraining.

#### Behavioural and physical indicators of progress within transition.

During transition, respondents most commonly monitored horses’ physical progress through visual assessment of gait (95%), the presence of injuries (95%), and assessment of the horse’s movement (92%), body condition score (78%), jumping performance (77%) and palpation of the horse’s body (63%) as well as visual assessment of the horse’s musculature (59%). Monitoring of gait using objective gait measurement tools (26%), heart rate (27%), horse’s bodyweight (31%), respiratory rate (39%), and assessment of the thoracic / back profile with a flexible ruler (39%) were less frequently assessed. Behavioural indicators assessed by retrainers to determine progress included evaluating a horse’s demeanour (98%), anxiety levels (98%), ability to cope mentally with work (98%), behaviour based on the rider’s or handler’s feedback (98%), attitude (97%), trainability (97%), happiness (97%), flight reaction (94%) and response to novel stimuli (82%). Few respondents (19%) utilized equipment or technology in monitoring the retraining process. The most common methods employed were using diary or written records (n = 8), photos (n = 6), video (n = 6), ride tracking applications (n = 5) and gait analysis software (n = 5) to assess horses’ progress.

#### Transition success.

**Fig 4:**
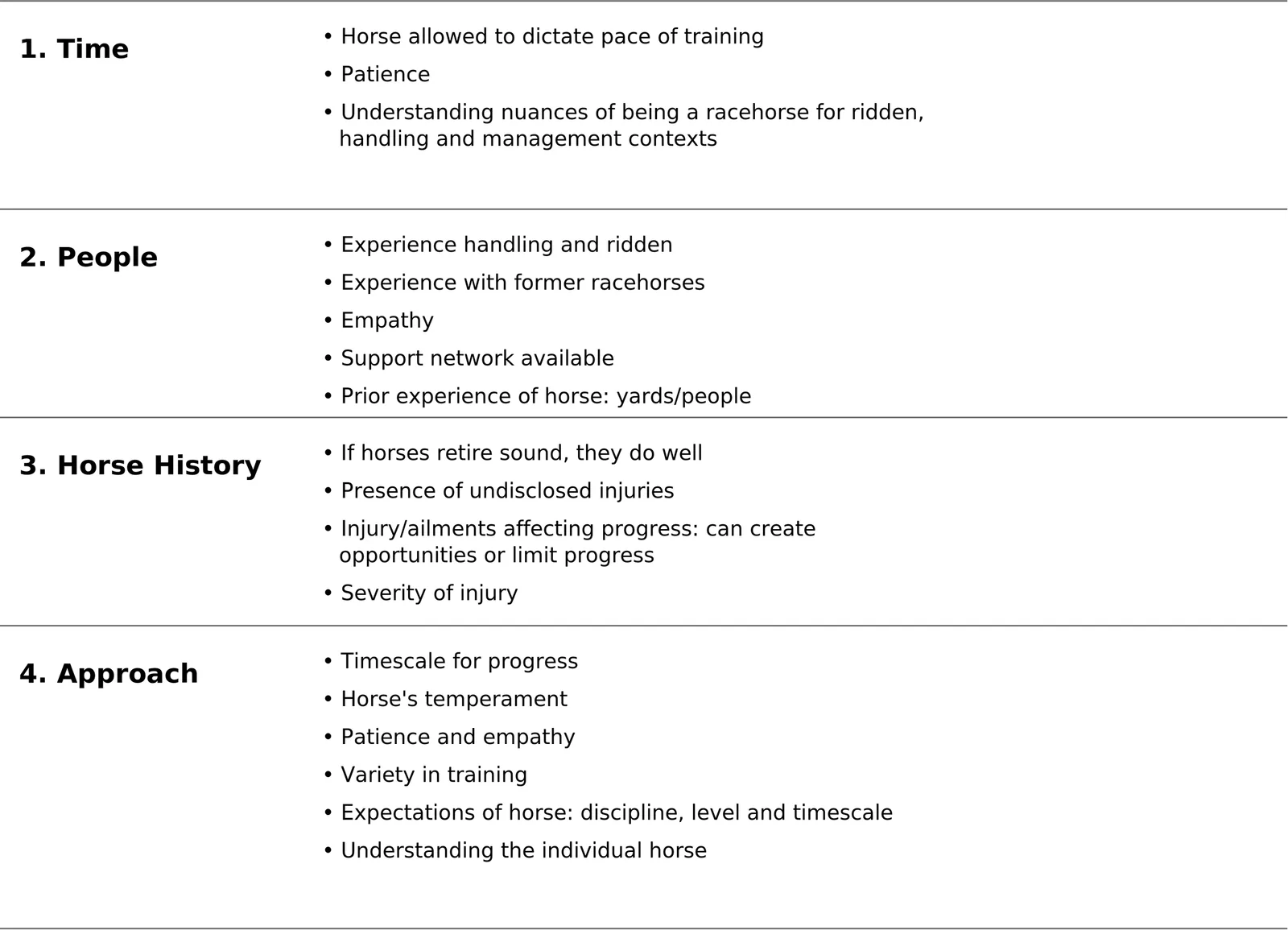
Respondent derived higher (bold) and lower order (italics) themes for which factors influence successful transition from racing. Respondents (n-100) were asked which factors influenced a successful transition for former racehorses to suitable second careers. Answers were coded using inductive conventional content analysis via a grounded theory approach to identify higher order themes: Time, People, History (horse) and Approach, and key lower order themes which underpinned these.

Respondents identified four key themes which they felt, based on their experiences, underpin successful retraining: 1) time, 2) people, 3) horse’s history, and 4) approach (Fig 4), with a a core focus on adapting transition to individual horse’s needs and experience, knowledge and understanding of racehorses and prior equestrian experience.

Three themes emerged from respondents that summarized when they felt a former racehorse had successfully transitioned to their second career: 1) [the horse was] happy and settled, 2) [the horse was] progressing forwards willingly in their work, and 3) [the horse had] the ability to cope and undertake their intended career; summed up by one respondent: “It is not an A to B linear process… They let me know when they are happy and comfortable in all different types of situations and with different riders able to ride them. They are so relaxed and happy in their own skin and are thriving. The retraining process though does not end with us, it will continue for many years and then although the horse is retrained well enough by us, we then have to find the people who are willing and capable to continue doing that as well for them.”

When asked how they selected a potential suitable second career for former racehorses, respondents identified five influential factors: 1) engagement: the horse enjoys and is relaxed in what they do, 2) intuition: the horse ‘tells’ me through training / work, 3) past history, 4) trial and error combined with [rider] understanding, 5) horse’s aptitude, attitude and ability to work [in a specific discipline]. The horse and their response to retraining were central in decision-making: “I go with what the horse is happy and capable of doing, as what I want can be quite different to what the horse is happy doing”.

Just over half of respondents (54%) felt it took them more than 8 weeks for them to gain sufficient insight into a horse to be able to identify suitable potential second careers; 23% felt they could tell within 5–6 weeks, and 20% could identify a suitable career in less than 5 weeks: “[Insight] Depends on each individual horse; [their] nature, attitude, trainability, talent, conformation, movement/action, stamina, previous injuries, breathing [issues].”

Four factors were rated as being very or extremely important when deciding a suitable second career for former racehorses: the horse’s new rider / owner (86%), the horse’s health (84%), the horse’s temperament (83%) and the horse’s trainability (81%) (S6 Table S3). The least influential factors, rated as not at all or slightly important only, were the horse’s level of training (48%) and the horse’s stable manners (31%). Most respondents (69%) had not had a horse returned to them after retraining, 20% had experienced a horse being returned rarely and 11% had experienced this occasionally. When horses had been returned, the reasons for this were a change of [owner] circumstances, horse unsuitability, because further help was required to enable a horse to settle to a new environment, or because the horse and rider match was unsuitable. The main challenges respondents faced when retraining former racehorses fall under two themes: 1) the horse and 2) perception [potential owners/keepers] (S7 Fig S1). When asked about tools or measures that could support the retraining process, respondents emphasized the importance of greater awareness and understanding of the individuality of former racehorses and its influence on transition.

### Delphi consultation results

Eighteen experts (13 retrainers and 5 musculoskeletal therapists) participated in Round 1 of the Delphi; ten musculoskeletal therapists participated in the intermediate focus groups. All participants met the expert eligibility criteria and were resident and involved in retraining in the United Kingdom. Round 2 had a retention rate of 67% (12 experts: 10 retrainers and 2 musculoskeletal therapists). Seven factors were agreed as essential to a successful transition (CVR > 0.7): effect of rider (0.96); effect of handler (1.0); change in feeding (forage/concentrate) (1.0); type of exercise (0.8); change of horse’s shape/musculature (0.8); posture/ how the horse holds itself (0.8); and change of turnout (0.8) (S8 Table S4; S9 Table S5).

There was higher agreement that basic flatwork (1.0), hacking in company (0.84) and working on varied terrain (0.7) should be included in a successful transition programme than hacking alone (0.5), and in-hand groundwork (0.5).

When monitoring progress, participants prioritized daily assessments of soundness (93.3%), monitoring of previous injury (93.3%) and monthly assessment of flatwork progression (93.3%), rating these as more important than weekly assessments of flatwork progression (66.7%), daily palpation of musculature or distal limbs (60.0%), weekly body condition scoring (53.3%), and weekly evaluation of muscle size or profile (53.3%). In contrast, heart rate and respiratory rate were infrequently monitored, with 40.0% and 26.7% of respondents, respectively, reporting that they would never assess these parameters.

### Features of a successful transition

Experts identified three overarching areas that underpinned a successful transition: 1) People; 2) Exercise Management; and 3) Treating each horse as an individual (Table 4). Participants consistently emphasised that the individuals involved in transition are critical to its success. Those supporting retraining were expected to possess strong skills in handling and interpreting Thoroughbred behaviour, to understand the differences between racing and post-racing environments, and be able to prepare horses accordingly, while adapting training in response to individual horse progress and needs:

**Table 4:** Themes that underpin former racehorse transition – Theme 1: People; Theme 2: Exercise Management; and Theme 3) Treating each horse as an individual.

**People**	*Handlers*	Individual skill set • Expertise: Able to ‘read’ horses • Calm, kind attitude • Understand Thoroughbreds • Capable to teach and train Thoroughbreds Support success • Train [horse] manners promoting safety • Understand racehorses and their management, utilize this to support successful transition to non-racing home
	*Riders*	Individual skillset • Experienced and empathetic • Knowledge and understanding of retraining • [Handler’s] Skills match horse’s needs Support success • Work horse ‘correctly’ in balanced frame • Understand different physical and behavioural needs from racehorse through retraining to transition to a riding horse • Capable of assessing horse progress
**Exercise** **Management**	*Adapt to the individual horse*	Consider prior history, injuries and fitness Utilize schooling / training plans Horse’s physicality and mentality drive process / progress
	*Exercise is essential*	Consistent approach Include new or expand exercise • Hacking • Flat work Targeted exercise approach to build muscle and develop new skills Integrate exercises to prepare / help horses adapt for future careers/ disciplines / new home Approach should match / suit horse’s physicality and mentality May include letdown / spelling periods (dependent on individual horse)
**Individualised Approach**	*Transition*	Tailor to individual horse and adapt to how they are coping physically and mentally Prior history, health, temperament and progress influences process – flexibility needed
	*Management*	May / may not include letdown / spelling period (dependent on individual horse} Change of routine • Integrate turnout / socialization • Evolve feeding regime to suit 2^nd^ career
	*Exercise*	Level / type dependent on horse history and health Have a plan: Frequency, duration, content within physical and behavioural limits Link exercise to new / second career
	*Regular assessment*	Evaluate progress monthly Evaluate behaviour: How is the horse coping? Assess general health Evaluate musculoskeletal health regularly: Lameness, pain and posture

Delphi panel experts were asked which factors underpin a successful transition for former racehorses to suitable second careers. Answers were coded using a ground theory approach to identify higher and lower order themes. For Theme 1: People, four higher order themes were identified, For Theme 2: Exercise Management, two higher order themes were identified, and for Theme 3: Individualized Approach, four higher order themes were identified> All are presented with their corresponding lower order themes where applicable.

*“It is essential that the rider is empathetic and understands retraining individuals…Riders must be able to understand how a Racehorse is ridden whilst in training to be able to help provide correct retraining for them. Retraining a racehorse is so different to riding a ‘normal’ riding horse!” Participant 1*

*“On the ground is just as important on their backs- the environment has to be safe, as do the horses… Important for manners and handling important for rehoming to leisure homes to ensure horse and handler safety.” Participant 3*

Participants identified adaptability to the individual horse, taking into account their history, capability, and fitness, as central components of exercise management. They also emphasized the importance of varied exercise, including activities that prepare horses for potential second careers by developing new skills, supporting muscular adaptation, and promoting mental engagement with new challenges:

*“As their retraining progresses, we always make sure that we work with them at the progress they feel most comfortable at. Some need to take more time to adjust as others prefer to keep busy.” Participant 4*

Consistently placing the individual horse at the centre of transition was rated as the most important aspect that determines the pace of transition, management, and exercise, complemented by regular progress assessment. Within this individualized approach, there were core areas that should be applied (albeit contextualized) to all horses to support a successful transition: The horse, its history and how they adapt and progresses through retraining;

*“We always listen to the horse. Some horses will openly show their anxiety as for others it can sometimes be very difficult to notice at first but may be brewing away inside them. If a horse lets us know that they are struggling to understand something or do something asked of them then instead of persisting, we will rethink about what they need and never force them out of them comfort zone both mentally and physically.” Participant 12*

Participants regularly evaluated factors they considered underpinned rehoming success including behaviour, manners, groundwork, hacking, handling; the presence of injury, lameness and pain, the horse’s body condition score, posture (how the horse holds itself statically and dynamically), muscle profile; and the ability to undertake basic flatwork skills and hack, and how settled the horse has become. They recommended utilizing allied professionals such as musculoskeletal therapists, veterinarians and coaches within assessment drawing on their expertise to evaluate progress usually monthly. However for some critical areas, such as health and injury, muscle development and posture, training and how the horse was coping in the environment, a reduced timeframe for assessment of between 2–4 weeks was advised. The individualized approach was summed up by one participant:

*“I’ve rehomed over 140 horses that have left the industry now and have come to find that all horses need a similar approach, yet with the flexibly in training / handling to accommodate for their own needs / training according to their racing history.” Participant 2*

Experts agreed that finding the right new owner for a horse was critical for a successful transition and acknowledged that this could be a challenging process. They looked to match people’s capability, experience and expectations to the horse’s capacity and used assessments visits and sourcing references from coaches, veterinarians, and other paraprofessionals to support this process. Participants also tried to identify which careers horses would be best suited to and to use this information to optimise a successful match but were also not afraid to say no and turn people down for individual horses if they felt they were unsuitable. As one participant identified:

*“You get a feel for someone as soon as you speak or message with them and then again as soon as the sit on a horse, by hearing what that person wants and needs, match it to the horse, see the person interact with the horse, ride him, and see where they will keep him. Get references from their vets and character refs.” Participant 6.*

## Discussion

This study aimed to determine what an optimal transition of former racehorses from racing to successful second careers should integrate drawing upon the insight of experienced retrainers and musculoskeletal therapists. While the survey and Delphi differed methodologically, their convergence around key themes strengthens confidence in the findings and highlights areas of consistency across stakeholder groups. Collectively, the results demonstrate that successful transition is underpinned by a horse-centred, flexible, and individualised approach, in which time, people, exercise management, and informed decision-making are critical. Importantly, both datasets reinforce that there is no single linear pathway from racing to a second career; instead, transition is a dynamic process shaped by each horse’s history, health, temperament, and future context.

A defining feature across both stages of the study was the recognition that transition timeframes are inherently variable. Respondents described transition periods ranging from a matter of weeks to more than two years, reflecting differences in horses’ physical condition on leaving racing, prior injury history, and psychological readiness to adapt to new environments. This aligns with previous work reporting that many retrainers are unable or unwilling to define minimum timeframes for retraining prior to rehoming, instead favoring open-ended, horse-led approaches [10]. Such findings challenge assumptions that transition can or should be standardized and instead support calls for flexibility in expectations placed on retrainers and owners. Across both the survey and Delphi consultation, successful transition was consistently framed not simply as the completion of retraining, but as securing an appropriate and sustainable match between the horse and its future owner or keeper. Positive matches align the horse’s physical and mental capacity with human expectations, thereby supporting long-term welfare and second career success regardless of discipline or role.

### An individualised and horse-centred approach

Across both stages of the study, focusing on the needs of the individual horse emerged as a unifying theme. Retrainers and musculoskeletal therapists emphasized that horses differ markedly in their physical resilience, learning speed, temperament, and adaptability, necessitating bespoke approaches to management, training, and progression. This finding aligns with broader welfare literature advocating for outcomes that support a horse’s “best life” rather than compliance with minimum welfare standards [10,43–45]. Stakeholders within the racing industry have similarly highlighted the limitations of one-size-fits-all approaches, arguing that attention to individual characteristics is essential to positive lifetime welfare outcomes.

The versatility of the Thoroughbred was repeatedly acknowledged across both stages of the study, with horses transitioning successfully into a wide range of ridden and non-ridden roles [4,7]. However, participants were clear that versatility should not be conflated with universal suitability. Not all horses are physically or mentally equipped to excel in every discipline, and inappropriate expectations risk compromising welfare and increasing the likelihood of breakdown or rehoming failure. The emphasis placed on individualization by participants is therefore reassuring from a welfare perspective, as it promotes realistic goal setting and mitigates pressure to force horses into unsuitable roles. At the same time, respondents acknowledged that genuinely horse-led approaches can be challenging to sustain economically, particularly where extended transition periods are required or where a quick turnaround for a former racehorse into a new home is the intended goal for economic reasons. This tension between ethical ideals and practical constraints underscores the importance of broader industry support for retraining and aftercare.

### Exercise management and physical adaptation

Exercise management emerged as a central pillar of successful transition across both the survey and Delphi consultation. While participants consistently stressed the need to tailor programmes to the individual horse, there was notable consensus around a core set of factors considered essential to transition, including feeding regime, rider and handler influence, type of exercise, monitoring and managing changes in the horse’s shape and posture, and provision of turnout. These areas reflect the substantial physiological shift horses undergo when moving from racing, characterized by high-intensity, high frequency work focused on racing performance attributes, e.g., galloping and jumping, to more varied forms of exercise required in second careers.

The energetic and musculoskeletal demands of most second careers are typically lower than those of racing, necessitating adjustments to both training volume and nutrition [46,47]. However, the point at which detraining ends and retraining begins is often blurred, particularly for horses leaving racing due to injury or following prolonged rest periods [48]. Participants described differing approaches to the inclusion of letdown, spelling or rest periods at the start of transition, with some advocating immediate continuation of work and others favoring weeks or months of turnout. Evidence suggests that both approaches carry potential benefits and risks. Letdown periods may facilitate adaptation to new management regimes and allow inflammatory processes to subside [43], yet abrupt reductions in workload may predispose some horses to back pain or hindlimb lameness when work is reintroduced [1,43].

Crucially, both the survey and Delphi highlighted that decisions around exercise and rest are heavily dependent on knowledge of the horse’s prior health and training history. Where such information is incomplete or unavailable, recognized as a common challenge identified by participants, managing transition effectively becomes more complex [41]. For example, horses that have developed compensatory musculature during racing may experience rapid atrophy during extended rest, necessitating careful reconditioning prior to ridden work to avoid pain-related behavioural issues [43]. These findings reinforce the need for improved transfer of health and management information at the point of retirement from racing [4].

Beyond decisions about rest, participants across both studies emphasized the importance of varied exercise during transition. Groundwork, hacking (alone and in company), work on varied terrain, and progressive flatwork were widely endorsed as foundational components of retraining. While some differences emerged between retrainers and musculoskeletal therapists, for example, in the perceived importance of pole work, an overarching principle was shared: exercise should prepare the horse physically and psychologically for the demands of its intended second career. This aligns with established principles of training and rehabilitation, which emphasize progressive loading, skill acquisition, and conditioning to reduce injury risk and support career longevity [17,49].

Monitoring physical adaptation during transition was also identified as essential, particularly in relation to changes in the horse’s shape and posture as work evolves. In sport horses, thoracic profile measurement has been shown to be a reliable method for tracking musculoskeletal change over time [50,51], and back pain is recognized as prevalent in Thoroughbreds [52]. Despite this, relatively few survey respondents reported routinely using objective measures such as thoracic profiling. This suggests an opportunity to integrate simple, low-cost assessment tools more systematically within transition, potentially prompting more timely saddle fitting and professional intervention.

### Behaviour, welfare, and psychological adaptation

While management factors such as feeding, turnout, and routine were frequently discussed by participants, both stages revealed that behavioural assessment is often implicit rather than explicit within transition practice. Stakeholders commonly referred to temperament, attitude, and how well a horse was “coping,” indicating reliance on subjective interpretation of behaviour to inform decision-making.

Behaviour is a critical indicator of welfare and quality of life [53], particularly during periods of significant environmental and social change such as retirement from racing. The emphasis on subjective behavioural assessment reflects broader trends within equestrian practice, where experience-based judgement often takes precedence over formalized behavioural metrics [17,54]. While experiential knowledge is undoubtedly valuable, the findings suggest that retrainers may benefit from greater support in recognizing and interpreting a wider range of behavioural indicators, including subtle signs of stress or discomfort. This is particularly pertinent given the strong links between behaviour, pain, and rider or handler safety [30,31]. Greater integration of behavioural expertise within transition, alongside veterinary, farriery, and musculoskeletal para-professional input, could enhance welfare outcomes and reduce the risk of mis-attributing behavioural issues to temperament rather than underlying physical causes.

### People, expectations, and matching horses to homes

Across both the survey and Delphi consultation, people emerged as fundamental to transition success. Participants consistently highlighted the importance of skilled, empathetic riders and handlers who understand the nuances of racehorse management and can adapt their approach accordingly. Equally critical was the process of matching horses to suitable owners or keepers following retraining. Successful transition was repeatedly defined not by the horse’s level of training alone, but by the compatibility between horse and human.

This finding resonates with existing literature indicating that rehoming success is influenced as much by human factors as by equine attributes [55]. Former racehorses are often attractive to prospective owners due to their affordability, athleticism, and perceived versatility [4,7,56], yet emotional decision-making can overshadow objective assessment of suitability [26,27]. Across the study, retrainers described active efforts to manage this risk through riding assessments, reference checks, and candid discussions about the realities of owning a former racehorse. Such practices are essential, given evidence that welfare issues within recreational equine populations are frequently linked to owner inexperience or misunderstanding [57].

Participants also emphasized the need for improved education and support for owners, particularly those new to Thoroughbreds. Traceability and access to accurate information about a horse’s history were also viewed as critical components of a robust aftercare system [4,9,58,59]. Encouragingly, initiatives within the UK aim to improve data sharing and ensure that horses retiring from racing are automatically connected with support networks such as Retraining of Racehorses (RoR) [4]. These developments align closely with the needs identified by participants across both sub-studies.

### Racehorse aftercare and industry responsibility

The findings of this study reinforce the growing recognition that racehorse aftercare is a shared industry responsibility, integral to racing’s social licence to operate [4,6,9,60]. Participants described a wide range of retraining pathways, from private individuals working one-to-one with horses to large, accredited rehoming centres and international programmes. Despite this diversity, there was consistent agreement that transition practices should align with accepted veterinary and welfare standards, even where this entails extended timeframes and financial cost [8,12].

However, respondents also expressed concern about the sustainability of current models, particularly where retrainers bear significant financial and emotional burdens. While funding mechanisms exist in some jurisdictions, such as mandatory levies or subsidized retraining programmes [9,12,61], not all horses pass through formal rehoming systems, leaving gaps in provision. These findings highlight the need for coordinated, multi-stakeholder approaches to ensure that all horses retiring from racing have access to appropriate support, regardless of their pathway.

### Multidisciplinary pathways to successful transition

Across both stages of the study, there is strong support for a multidisciplinary approach to transition. Although not always explicitly framed as such by participants, the involvement of veterinarians, farriers, musculoskeletal therapists, saddle fitters, and coaches was frequently described as integral to managing complex cases. Multidisciplinary team (MDT) models are well established in human healthcare and increasingly advocated within animal health and elite equine sport [62–73]. The emphasis placed on regular assessment, adaptability, and collaboration within this study suggests that MDT principles are already informally embedded within transition practice.

**Fig 5:**
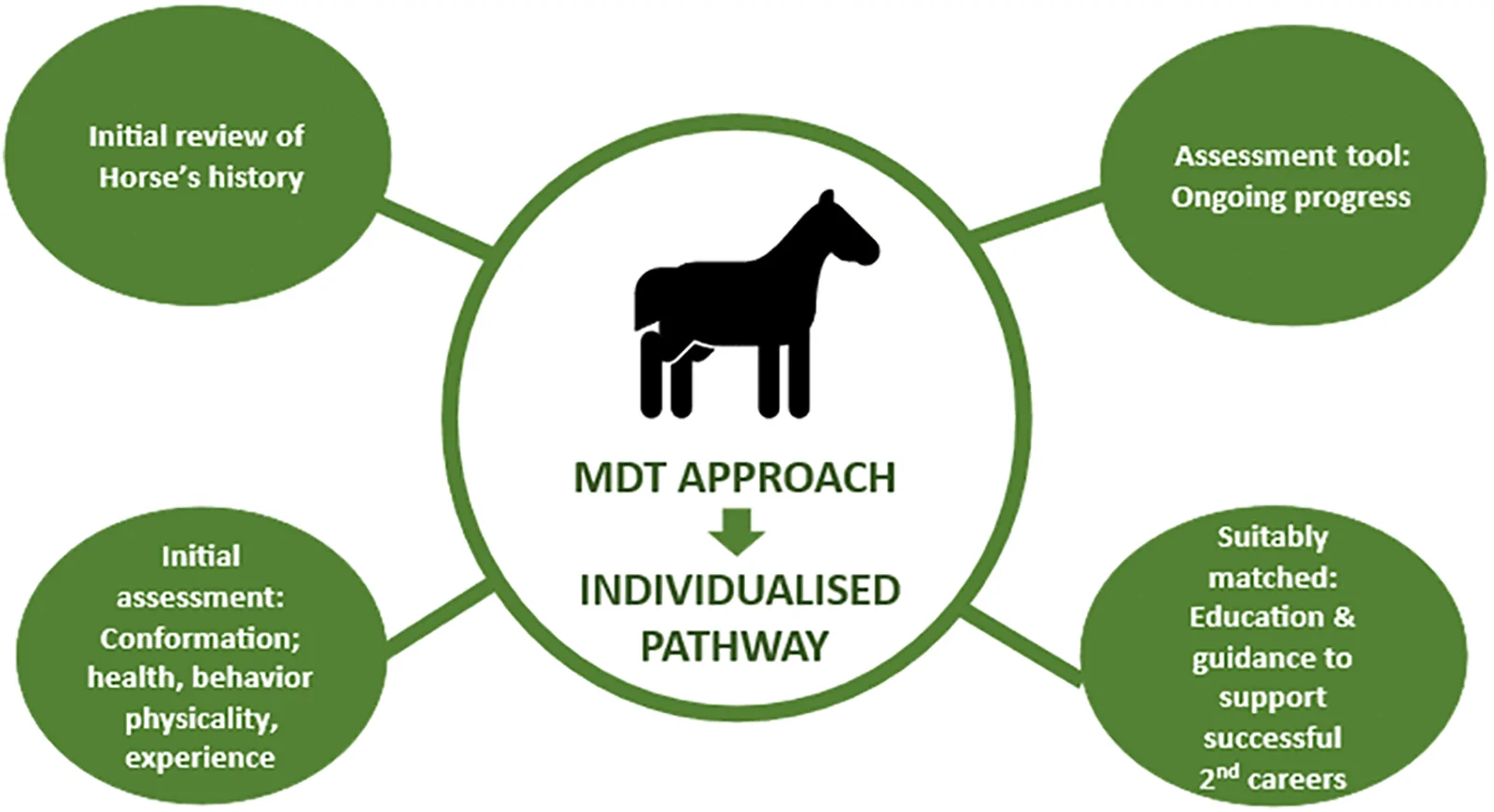
Transition triage: utilising a multidisciplinary team approach to determine suitable individualised pathways that result in a successful transition from racing to future careers. Drawing on common themes from the results a framework which could be applied to inform transition of former racehorses. MDT: multidisciplinary team.

The proposed transition triage framework (Fig 5) builds on this implicit practice by providing a structured model that places the horse at the centre of decision-making. By integrating review of history, initial assessment, ongoing monitoring, and owner guidance, such frameworks have the potential to support consistent, evidence-informed pathways to second career success while retaining the flexibility required to meet individual needs.

### Study limitations

Several limitations should be acknowledged. The study focused on the initial phase of transition from racing to a first second career and did not examine long-term outcomes beyond rehoming, nor pathways into breeding careers. Success was primarily evaluated from a health and performance perspective, and broader welfare considerations relating to housing, management, and long-term suitability of second careers warrant further investigation. Both the survey and Delphi relied on self-reported data, which may be subject to respondent bias. Additionally, most participants were based in the UK, Australia, or New Zealand, and findings may not fully reflect practices in other racing jurisdictions. Furthermore, cultural norms and industry practices and opinions may vary across different sociocultural contexts, which may also limit the generalisability of these findings.

As with all Delphi studies, outcomes are shaped by the expertise of participating panel members. Although inclusion criteria were applied to ensure relevant experience, the absence of veterinarians as a distinct expert group may have limited perspectives in some areas. The intermediate focus group participants were recruited through professional networks and musculoskeletal-focused social media groups, with the aim of capturing a range of professional perspectives. However, all five respondents were ACPAT Chartered Physiotherapists, an outcome of convenience sampling rather than intentional selection. While this may limit the diversity of viewpoints represented in the qualitative phase, the focus group findings were used to refine and contextualize domains rather than to establish consensus, which was determined through the broader Delphi panel comprising both physiotherapists and retrainers. Future studies may benefit from targeted recruitment strategies to ensure representation across professional groups within qualitative components.

Attrition across Delphi rounds was observed but remained within accepted thresholds. A 70% agreement threshold was adopted for this study, consistent with commonly accepted standards in Delphi methodology where thresholds typically range from 51% to 80% depending on study objectives [28]. For a Round 2 panel size of 12, a CVR critical value of 0.70 was applied across all rounds. Our approach therefore balances methodological rigour with practical considerations of panel size, while remaining consistent with established standards for Delphi consensus studies. This threshold is more conservative than Lawshe’s [39] original recommendation of 0.56 for a panel of 12, and aligns more closely with the adjusted critical values proposed by Ayre and Scally [74], who suggest that a panel of 12 requires a minimum of 10 members rating an item as essential to demonstrate validity beyond chance. This would equate to an essential consensus threshold of >0.8, which the impact of rider/s, handler/s and change in feeding would exceed. Future research employing qualitative methodologies and broader stakeholder inclusion would provide valuable depth and further validate these findings.

## Conclusions

This study provides an expert-informed synthesis of factors underpinning the successful transition of former racehorses into second careers. The results demonstrate that effective transition is not a linear or time-bound process, but one that must remain flexible, horse-centred, and responsive to individual physical, behavioural, and historical factors. Across both study stages, people, exercise management, and individualization emerged as the core determinants of positive outcomes. Consensus was achieved on seven essential components of transition, emphasizing the critical influence of rider and handler expertise, feeding and turnout practices, and the management of posture, musculature, and exercise type. These elements reflect the substantial physical and psychological adaptations required when horses move from racing to new roles and reinforce the importance of addressing mental wellbeing alongside musculoskeletal health. Successful transition was consistently defined not by the speed of retraining or level of performance achieved, but by the horse’s ability to cope, settle, and be matched appropriately to a future owner and career.

Collectively, these findings support the development of evidence-informed, multidisciplinary transition pathways that prioritise individual horse needs while acknowledging practical and economic constraints. Improving access to professional expertise, enhancing owner education, and strengthening information transfer at the point of retirement from racing may further support sustainable second careers and long-term welfare.

## Supporting information

S1 File Online survey: Transition of former racehorses / off the track TBs. (DOCX)

S2 File CREDES checklist for delphi studies. Guidance on conducting and REporting DElphi Studies (CREDES).
(DOCX)

S3 File Delphi Stage 1 survey: Transition from racehorse to second careers. (DOCX)

S4 Table S1: Frequency of factors which respondents use when selecting former racehorses for retraining. (DOCX)

S5 Table S2: Frequency of factors which influence how long respondents will retrain a former racehorse for. (DOCX)

S6 Table S3: Respondent rating of importance of factors when deciding which discipline or activity a former racehorse is suited for. (DOCX)

S7 Fig S1: Key challenges respondents identify when rehoming former racehorses. (DOCX)

S8 Table S4: Delphi Round 1 expert consensus overview. (DOCX)

S9 Table S5: Delphi Round 2 expert consensus overview. (DOCX)
