# Correction: Transforming retraining: Supporting racehorses to have successful second careers

**DOI:** 10.1371/journal.pone.0360303

**Published:** 2026-10-07

**Authors:** Jane M. Williams, Saranna Jordan, Laura Friend, Emily Kay, Matilda Edmunds, Kathryn Nankervis, Jessica Yeung, Gillian Tabor

The seventh author’s name is spelled incorrectly. The correct name is: Jessica Yeung.
