# Supplementary material for: Transforming retraining: Supporting racehorses to have successful second careers
Source: PLoS One. 2026 Aug 19;21(8):e0355509. doi: 10.1371/journal.pone.0355509 (PMC13489413; doi:10.1371/journal.pone.0355509)
Supplement: Supplementary file 1 [file pone.0355509.s001.docx]

S1 Online survey: Transition of former racehorses / off the track TBs

Introduction

We would like to invite you to take part in a survey to determine the key behavioral and physical factors that affect racehorses when they leave training, and which can influence a successful transition to a second career. Retraining is arguably fundamental or critical to a horse's success after racing. We hope the results of the survey will help us inform decisions on retraining practice and optimize ‘performance in early retraining’ for racehorses entering their second careers.

The survey should not take more than 10 minutes to complete. To be eligible to take part, you should be experienced in retraining of Thoroughbred racehorses and be over 18 years of age. Please note that all information which is collected during the course of the research will be kept securely. The survey is anonymous and collects no identifiable personal data about you, so that you cannot be recognized from it. It is up to you to decide whether or not to take part in this research. By filling out this questionnaire you are indicating your voluntary informed consent for your results to be included in data analysis. You are free to withdraw without giving a reason up the point of pressing submit at the end of the survey.

This survey is a Hartpury University and Hong Kong Jockey Club research project (Ethics number: 2022-14). The research team comprises: Dr Jane Williams, Dr Gillian Tabor, Dr Kathryn Nankervis, Laura Friend, Saranna Jordan, Steph Evans and Jess Williams. The project is being funded by the Hong Kong Jockey Club.

It is our intention to present the results to the Hong Kong Jockey Club. We also aim to present them at a relevant conference, publish them in an appropriate journal, and disseminate them to a wider audience via articles in the equestrian lay press and industry dissemination.

If you have any questions or comments regarding this survey please use the box provided at the end of the survey or email Dr Jane Williams at jane.williams@hartpury.ac.uk Your views and experiences are key to make this project a success, we really value your input and thank you for taking the time to complete the survey.

Consent

It is up to you whether or not you choose to take part in this research. By filling out this questionnaire, you are indicating your voluntary informed consent for your results to be included in data analysis.

You are free to withdraw without giving a reason up to the point of pressing submit at the end of the survey.

I acknowledge that my participation in the study is voluntary, I am over 18 years of age, and that I provide consent for data provided to be used for research purposes.
 Yes

Section 1 **Your background and experience**

Q1 How long ago did you start retraining former racehorses / off the track thoroughbreds (OTTBs)?

- Less than 1 year
- 1-2 years
- 3-5 years
- 5-10 years
- Over 10 years

Q2 How many former racehorses / OTTBs have you rehomed or retrained during this time?

- 1-2 horses
- 3-5 horses
- 5-10 horses
- 10+ horses

Q3 Where are you located?

- Great Britain (including Ireland)
- Europe
- USA or Canada
- Australia or New Zealand
- Asia: China, Hong Kong or Japan
- Other, please state: (6) __________________________________________________

Q4 We would like to know more about you, please select all options below which describe you (tick all that apply):

- Have an equestrian qualification
- Have a veterinary qualification
- Have a coaching qualification
- I am an equine musculoskeletal therapist (e.g., physiotherapist, chiropracter, masseur, body worker)
- I have practical experience in the equine industry
- I have a personal history in racing
- I hold a professional role in the racing industry
- I hold a professional role in the broader equestrian industry

Q5 Please select the option which best describes your involvement in retraining former racehorses / OTTBs:

- Own / work in a private retraining yard
- Own / work in a TB retraining yard
- Retrain individual TBs on a 1-2-1 basis, professionally
- Retrain individual TBs on a 1-2-1 basis, on a personal level
- Work with a trainer to retrain racehorses
- Other, please state below: __________________________________________________

Q6 Which disciplines do you retrain former racehorses / OTTBs for? Please tick all that apply.

- Leisure riding / hacking / trail riding
- Equine assisted activities
- Showing
- Endurance riding
- Show jumping
- Dressage
- Eventing
- Hunting / Trail Hunting
- Polo
- Horseball
- Team Chasing
- Hunter Jumper
- Riding School
- Other, please state below: _________________________________________________

Q7 What are your reasons for retraining former racehorses / OTTBs? Please tick all that apply.

- Approached by industry / owner / keeper / trainer
- Business
- Employment
- To have your own horse
- Dedicated retrained racehorse competition opportunities
- Other competitive opportunities
- Provide a good quality of life
- Individual attachment to a specific racehorse
- Other, please state below: __________________________________________________

- If you would like to tell us more, please do so below:

________________________________________________________________

Section 2 **Behavioural and physical challenges to racehorses leaving training**

Q8 How much do you feel that changes within the following factors can influence a horse's physical condition when starting their retraining?

|  | Always | Very Likely | Likely | Unlikely | Very Unlikely | Never | Unsure |
| --- | --- | --- | --- | --- | --- | --- | --- |
| Amount of exercise / training |  |  |  |  |  |  |  |
| Training / exercise / yard routine |  |  |  |  |  |  |  |
| Time spent in the paddock / free exercise |  |  |  |  |  |  |  |
| Time spent in the stable |  |  |  |  |  |  |  |
| Different types of work |  |  |  |  |  |  |  |
| Working on different surfaces |  |  |  |  |  |  |  |
| Bridle / Bit used |  |  |  |  |  |  |  |
| Saddle used |  |  |  |  |  |  |  |
| Use of training aids |  |  |  |  |  |  |  |
| Rider position due to the saddle |  |  |  |  |  |  |  |
| Different posture / outline / frame due to new discipline requirements |  |  |  |  |  |  |  |
| Shoeing / trimming routine |  |  |  |  |  |  |  |
| Rider weight |  |  |  |  |  |  |  |
| Change in the horse's shape, e.g., weight, muscle, body condition |  |  |  |  |  |  |  |

Q9 Are there any other factors that you feel influence a horse's physical condition after leaving racing or starting their retraining that we have not mentioned? Please detail below.

Q10 How much do you feel changes in the following factors can influence a horse's behaviour when starting their retraining?

|  | Always | Very Likely | Likely | Unlikely | Very Unlikely | Never | Unsure |
| --- | --- | --- | --- | --- | --- | --- | --- |
| Change of feeding concentrates e.g., grain, hard food |  |  |  |  |  |  |  |
| Change of feeding forage e.g. hay, haylage, alfafa, grass |  |  |  |  |  |  |  |
| Change of turnout / free exercise |  |  |  |  |  |  |  |
| Time spent in the stable |  |  |  |  |  |  |  |
| Change from working in a group to working alone |  |  |  |  |  |  |  |
| Frequency of exercise |  |  |  |  |  |  |  |
| Type of exercise |  |  |  |  |  |  |  |
| Rider / handler |  |  |  |  |  |  |  |
| Change of environment - other horses |  |  |  |  |  |  |  |
| Change of environment - climate |  |  |  |  |  |  |  |
| Change of environment - yard |  |  |  |  |  |  |  |
| Change of daily routine from racing |  |  |  |  |  |  |  |
| Change in transportation method e.g., trailer / float, lorry |  |  |  |  |  |  |  |
| Increased /decreased changes in opportunities for contact with other horses ( |  |  |  |  |  |  |  |

Q11 Are there any other factors that you feel influence a horse's behaviour after leaving racing or starting their retraining that we have not mentioned? Please detail below.

________________________________________________________________

________________________________________________________________

________________________________________________________________

________________________________________________________________

________________________________________________________________

Q12 How much do you feel the following factors, which may affect racehorses in training, could impact the success of horses when retraining, and in their future careers?

|  | Always | Very Likely | Likely | Unlikely | Very Unlikely | Never | Unsure |
| --- | --- | --- | --- | --- | --- | --- | --- |
| Muscle pathology / disease |  |  |  |  |  |  |  |
| Chronic lameness |  |  |  |  |  |  |  |
| Intermittent lameness |  |  |  |  |  |  |  |
| Poor performance |  |  |  |  |  |  |  |
| Breathing problems / airway disease |  |  |  |  |  |  |  |
| EIPH / bleeders |  |  |  |  |  |  |  |
| Cardiovascular disease |  |  |  |  |  |  |  |
| Gastric Ulcers |  |  |  |  |  |  |  |
| Muscular back pain |  |  |  |  |  |  |  |
| Skeletal back pain |  |  |  |  |  |  |  |
| Pelvic / lumbosacral / sacroiliac (SI) region pain |  |  |  |  |  |  |  |
| Limb - bone or joint issues / disease |  |  |  |  |  |  |  |
| Limb - tendon or ligament injury |  |  |  |  |  |  |  |
| Foot pain |  |  |  |  |  |  |  |

Q13 Reflecting on your experience, are there any other factors which impact the success of former racehorses / OTTBs during retraining and / or in their future careers? Please detail below.

________________________________________________________________

Section 3 **Stages and duration of training**

Q14 For horses which have left racing without injury, when, on average, do you believe is the most appropriate time to start the retraining process?

- Immediately after retirement
- 4 weeks let down / spell / rest / time out of work
- 6-8 weeks let down / spell / rest / time out of work
- 9-12 weeks let down / spell / rest / time out of work
- 13-16 weeks let down / spell / rest / time out of work
- Between 4 and 6 months let down / spell / rest / time out of work
- Longer than 6 months let down / spell / rest / time out of work

Q15 What is your typical retraining process? Please tick all that apply.

|  | Would always include in a retraining programme | Would aim to include but it would depend on the individual horse | Would not include in a retraining programme |
| --- | --- | --- | --- |
| Long lining |  |  |  |
| Lunging |  |  |  |
| Ground work / in hand training |  |  |  |
| Hacking |  |  |  |
| Basic flat work e.g., simple transitions, easy turns and circles |  |  |  |
| More advanced flat work e.g., direct transitions, lateral work, adjusting stride length |  |  |  |
| Leading from other horses |  |  |  |
| Work in open spaces |  |  |  |
| Work on varied terrain |  |  |  |
| Pole work |  |  |  |
| Grid work |  |  |  |
| Jumping: show jumps |  |  |  |
| Jumping: fixed obstacles / cross country |  |  |  |
| Experiencing different venues / facilities / locations |  |  |  |
| Competition experience |  |  |  |

Q16 Please indicate how many weeks after the start of retraining you would introduce the activities listed:

- Long lining (______________________________________________
- Lunging _________________________________________________
- Ground work / in hand training ____________________________
- Basic flat work e.g., simple transitions, easy turns and circles ___
- More advanced flat work e.g., direct transitions, lateral work and adjusting stride length ___________________________________________________________
- Leading from other horses _________________________________
- Work in open spaces _______________________________________
- Work on varied terrain _____________________________________
- Pole work	_______________________________________________
- Grid work	________________________________________________
- Jumping: show jumps _______________________________________
- Jumping: fixed obstacles / cross country ________________________
- Experiencing different venues / facilities / locations ______________
- Competition experience ______________________________________

Q17 What is the average amount of time you would expect to have a former racehorse / OTTB in your care for retraining for?

- 0-3 months
- 4-6 months
- 6-12 months
- 12+ months
- up to 2 years
- 2+ years

Section 4 **Behavioural and physical indicators of progress in retraining**

Q18 Do you measure any of the following physical factors to monitor horses' progress in retraining?

|  | Yes | No |
| --- | --- | --- |
| Body weight (on weigh bridge) |  |  |
| Body condition score |  |  |
| Heart rate |  |  |
| Respiratory rate |  |  |
| Muscle size |  |  |
| Thoracic / back profile with flexible ruler |  |  |
| Gait: visual observation |  |  |
| Gait: objective measurement |  |  |
| Palpation |  |  |
| Movement |  |  |
| Jumping |  |  |
| Injuries |  |  |
| Other please state below: |  |  |

Q19 Do you measure any of the following behavioural factors to monitor horses' progress in training?

|  | Yes (1) | No (2) |
| --- | --- | --- |
| Attitude |  |  |
| Demeanour |  |  |
| Flight reaction / instinct |  |  |
| Happiness |  |  |
| Anxiety levels |  |  |
| Response to novel stimuli |  |  |
| Ability to cope with work |  |  |
| Rider / handler feedback |  |  |
| Trainability |  |  |
| Other, please state below: |  |  |

Q20 Do you use any equipment or technology to record or monitor horse's progress? If yes, please tell us which use below:

________________________________________________________________

________________________________________________________________

Q21 How do you know a former racehorse / OTTB is successfully retrained?

________________________________________________________________

________________________________________________________________

Section 5 **Decision-making factors that inform the selection of a suitable future career for individual horses**

Q22 How do you initially select which thoroughbreds to retrain? Please select all that apply:

|  | Always | Very Likely | Likely | Unlikely | Very Unlikely | Never |
| --- | --- | --- | --- | --- | --- | --- |
| Intuition |  |  |  |  |  |  |
| Emotional attachment |  |  |  |  |  |  |
| Assessment tool / matrix / system |  |  |  |  |  |  |
| Conformation |  |  |  |  |  |  |
| Prior health history |  |  |  |  |  |  |
| Physical assessment |  |  |  |  |  |  |
| Behaviour assessment |  |  |  |  |  |  |
| Temperament assessment |  |  |  |  |  |  |
| Breeding |  |  |  |  |  |  |
| Cost |  |  |  |  |  |  |
| Other, please state below: |  |  |  |  |  |  |

Q23 Which of the following factors influence the length of time you would retrain a former racehorse / OTTB for?

|  | Always | Very Likely | Likely | Unlikely | Very Unlikely | Never |
| --- | --- | --- | --- | --- | --- | --- |
| Horse's physical health |  |  |  |  |  |  |
| Horse's prior injuries |  |  |  |  |  |  |
| Horse's mental wellbeing |  |  |  |  |  |  |
| Horse's suitability for the selected discipline |  |  |  |  |  |  |
| Horse's trainability |  |  |  |  |  |  |
| Affordability |  |  |  |  |  |  |
| Time constraints |  |  |  |  |  |  |
| Suitability of premises / livery |  |  |  |  |  |  |
| Other, please state below: |  |  |  |  |  |  |

Q24 How do you select potential suitable second careers for the former racehorses / OTTBs you have retrained?

________________________________________________________________

________________________________________________________________

________________________________________________________________

Q25 At what point in retraining do you believe you can identify or have an insight into the horse's most suitable second career option or pathway?

- 0-2 weeks
- 3-4 weeks
- 5-6 weeks
- 7-8 weeks
- 8+ weeks

Q26 Please rate how important you feel the following factors are when deciding which discipline or activity a former racehorse / OTTB is suited for.

|  | Not at all important | Slightly important | Moderately important | Very important | Extremely important |
| --- | --- | --- | --- | --- | --- |
| Conformation |  |  |  |  |  |
| Temperament / personality |  |  |  |  |  |
| Rideability |  |  |  |  |  |
| Trainability |  |  |  |  |  |
| Athleticism |  |  |  |  |  |
| Level of training |  |  |  |  |  |
| Health |  |  |  |  |  |
| Able to be ridden alone |  |  |  |  |  |
| Able to be ridden in company |  |  |  |  |  |
| Stable manners |  |  |  |  |  |
| Interaction with humans |  |  |  |  |  |
| New rider's / owner's experience |  |  |  |  |  |

Q27 How often do horses come back into your care after rehoming?

- Never
- Rarely
- Occasionally
- Regularly
- All the time

Q28 What are the key reasons a horse is returned to you?

________________________________________________________________

Q29 What are the main challenges you face when retraining racehorses?

________________________________________________________________

Q30 Are there any tools or measures (which currently exist or could be developed), which would help you increase your success in retraining former racehorses / OTTBs?

________________________________________________________________

Feedback. Thank you for completing the survey. If you have any further comments on retraining former racehorses / OTTBs, or have any feedback on the survey, please include this below.

________________________________________________________________

Delphi Stage 1 Survey: Transition from racehorse to second careers

Introduction

We would like your help as an expert professional retrainer of former racehorses to find out more about retraining methods and practices in the UK, investigating the common features and key stages of successful early retraining. Our aim is to develop a consensus for good practice and to describe common retraining practices including the identification of physical and behavioral characteristics used to monitor progress across the retraining period. Thank you for your support of this project.

This process is likely to include 3 rounds of surveys, one every 2-3 weeks across October and November. To be successful, it is important that your opinion is captured and shapes the content to make this an interactive and iterative process. This initial Stage 1 questionnaire should not take more than 10 minutes to complete.

Please note that all information which is collected about you during the course of the research will be kept securely. The survey is anonymous and collects no identifiable personal data about you, so that you cannot be recognised from it; however, as we will be contacting the same group of people who are taking part in this project, we will keep a record of who has replied. None of your answers will be linked to your details.

It is our intention to present the results within a final consensus statement, which will be presented at a relevant conference, published in an appropriate journal, and disseminated to a wider audience via articles in the equestrian lay press, as well as being shared with the Hong Kong Jockey Club, as the funders of this project.

This survey is a Hartpury University and Hong Kong Jockey Club research project (ethics number: ETHICS2022-14 ). The research team comprises: Dr Jane Williams, Dr Gillian Tabor, Dr Kathryn Nankervis, Laura Friend, Saranna Jordan, Jess Williams and Steph Evans.  

 If you have any questions or comments regarding this survey please use the box provided at the end of the survey or email Dr Gillian Tabor at gillian.tabor@hartpury.ac.uk
Thank you again for supporting our work.

Consent

It is up to you to decide whether or not to take part in this research. By filling out this questionnaire you are indicating your voluntary informed consent for your results to be included in data analysis. You are free to withdraw without giving a reason up the point of pressing submit at the end of the survey. I acknowledge that my participation in the study is voluntary, I am over 18 years of age, and that I provide consent for the data provided to be used for research purposes.

- Yes I provide consent

Q1 Please can you tick which role best describes your involvement in retraining:

- Trainer
- Veterinarian
- Musculoskeletal therapist

Q2 How many years have you / your facility / your team been involved with retraining former racehorses?

- Less than 1 year
- 1 to 3 years
- 3 to 5 years
- 5 to 10 years
- Longer than 10 years

Q3 How many former racehorses (approximately) have you retrained / been involved with retraining?

- 1 to 5 racehorses
- 6 to 10 racehorses
- 11 to 15 racehorses
- 16 to 20 racehorses
- 20 to 50 racehorses
- More than 50 racehorses

Q4 Based on your experience, please rate how influential you feel the following areas are within a successful racehorse retraining programme? Please select the answer which most closely represents your view:

|  | Always influential (1) | Important but not always influential (2) | Not influential (3) |
| --- | --- | --- | --- |
| Amount of exercise (1) |  |  |  |
| Amount of training (2) |  |  |  |
| Type of exercise (3) |  |  |  |
| Different posture (e.g., how the horse holds itself) (4) |  |  |  |
| Change in the horse's shape (e.g., musculature) (5) |  |  |  |
| Change of daily routine from racing (6) |  |  |  |
| Change of feeding forage and feeding concentrates (7) |  |  |  |
| Change of turnout (8) |  |  |  |
| Amount of free exercise (not ridden or part of training / exercise programme) (9) |  |  |  |
| The rider/s (10) |  |  |  |
| The handler/s (11) |  |  |  |
| Type of training: flat, national hunt or unraced (12) |  |  |  |
| Horse's temperament (13) |  |  |  |

Q5 Are there any other physical or behavioural factors which you feel influence the success of a racehorse's retraining programme? Please describe these below.

________________________________________________________________

Q6 How do you measure/monitor behavioural factors during retraining? Examples of these factors are horses’ demeanour, anxiety levels, ability to cope mentally with work and their trainability. Please describe below.

________________________________________________________________

Q7 Based on your experience, please rate how influential you feel the following areas related to horse health and injury are within a successful racehorse retraining programme? Please select the answer which most closely represents your view:

|  | Always influential (1) | Important but not always influential (2) | Not influential (3) |
| --- | --- | --- | --- |
| Muscle issues (1) |  |  |  |
| Skeletal back pain (2) |  |  |  |
| Pelvic \| Sacro-iliac (SI) \| Thoraco-lumbar (TL) pain (3) |  |  |  |
| Persistent lameness (4) |  |  |  |
| Intermittent lameness (5) |  |  |  |

Q8 Are there any other health or injury related factors which you feel influence the success of a racehorse's retraining programme? Please describe these below.

________________________________________________________________

Q9 Based on your experience, please rate how essential you feel the following activities are within a successful racehorse retraining programme? Please select the answer which most closely represents your view:

|  | Always essential | Important but not essential | Not essential |
| --- | --- | --- | --- |
| Pole work |  |  |  |
| Grid work |  |  |  |
| In-hand / ground work |  |  |  |
| Hacking (alone) |  |  |  |
| Hacking (in company) ( |  |  |  |
| Basic flatwork e.g., simple transitions |  |  |  |
| Advanced flatwork e.g., lateral work |  |  |  |
| Work in open spaces |  |  |  |
| Work on varied terrain |  |  |  |
| Work on the lunge |  |  |  |

Q10 Are there any other activities which you feel are essential to include in a successful racehorse retraining programme? Please describe these below.

________________________________________________________________

Q11 For the activities listed below, approximately how many weeks after the start of the retraining process would you introduce them within your retraining plan? Please insert as whole weeks, e.g., if you would introduce hacking 2 weeks after the start of retraining, you would insert '2'.

- Pole work	__________________________________________________
- Grid work	__________________________________________________
- In hand / ground work _______________________________________
- Hacking (alone) _____________________________________________
- Hacking (in company) ________________________________________
- Basic flatwork e.g., simple transitions ___________________________
- Advanced flatwork e.g., lateral work ___________________________
- Work in open spaces _________________________________________
- Work on varied terrain _______________________________________

Q12 What is the average amount of time you would expect to have a former racehorse in your care for retraining for? Please write your answer below as a whole number and state if this is weeks, months or years e.g., 4 weeks.

________________________________________________________________

Q13 How often would you use the following factors to assess a racehorse’s progress during the retraining process?

|  | Never | Daily | Weekly | Monthly | Every 2 months | Every 6 months | Annually |
| --- | --- | --- | --- | --- | --- | --- | --- |
| Body condition score |  |  |  |  |  |  |  |
| Level of soundness |  |  |  |  |  |  |  |
| Monitoring of previous injury |  |  |  |  |  |  |  |
| Progress in jumping |  |  |  |  |  |  |  |
| Progress in flatwork |  |  |  |  |  |  |  |
| Muscle size / profile |  |  |  |  |  |  |  |
| Palpation: feeling through/over muscles and/or lower limb |  |  |  |  |  |  |  |
| Respiratory rate at rest or exercise |  |  |  |  |  |  |  |
| Heart rate at rest or exercise |  |  |  |  |  |  |  |
| Thoracic back profile (top line profile) |  |  |  |  |  |  |  |

Q14 How do you know when a former racehorse is successfully retrained? Please describe below.

________________________________________________________________

Q15 What would make you change from an expected/standard retraining plan for a former racehorse? Please describe below.

________________________________________________________________

Q16 What are the main challenges you face as a retrainer of racehorses? Please describe below.

________________________________________________________________

Q17 Reflecting on your experience, are there any other factors that you feel impact the success of former racehorses in retraining or within their future careers? Please describe below.

________________________________________________________________

Comments If you would like to add any further comments on retraining racehorses or the Stage 1 survey, please include these below.

________________________________________________________________
