# Supplementary material for: Transforming retraining: Supporting racehorses to have successful second careers
Source: PLoS One. 2026 Aug 19;21(8):e0355509. doi: 10.1371/journal.pone.0355509 (PMC13489413; doi:10.1371/journal.pone.0355509)
Supplement: Supplementary file 2 [file pone.0355509.s002.docx]

**S3 CREDES Checklist for Delphi Studies**

**Guidance on Conducting and REporting DElphi Studies (CREDES)**
(Junger et al., 2017)

| **CREDES Item** | **Description** | **Location in Manuscript** |
| --- | --- | --- |
| **1. Study aim** | Clear statement of the aim of the Delphi study | Introduction; Methods Section 2.2 |
| **2. Rationale for Delphi method** | Explanation for selecting a Delphi approach | Methods Section 2.2 |
| **3. Study context** | Description of how the Delphi fits within the overall study design | Methods Sections 2.1 and 2.2 |
| **4. Definition of expert panel** | Criteria used to define participant expertise | Methods Section 2.2.1 |
| **5. Eligibility criteria** | Inclusion criteria for panel participation | Methods Section 2.2.1 |
| **6. Recruitment strategy** | How participants were identified and invited | Methods Section 2.2.1; S1 Appendix |
| **7. Panel size** | Number of participants invited and participating | Methods Section 2.2.1; Results |
| **8. Panel composition** | Professional background of panel members | Methods Section 2.2.1 |
| **9. Anonymity of responses** | Whether participant responses were anonymous | Methods Section 2.2 |
| **10. Iterative design** | Use of multiple rounds to refine consensus | Methods Sections 2.2.2 and 2.2.4 |
| **11. Number of Delphi rounds** | Total number of rounds conducted | Methods Section 2.2 |
| **12. Feedback between rounds** | Description of controlled feedback provided | Methods Section 2.2 |
| **13. Development of survey items** | How survey questions and domains were generated | Methods Sections 2.1 and 2.2 |
| **14. Participant input** | Opportunity for participants to suggest additional items | Methods Section 2.2.2 |
| **15. Definition of consensus** | A priori definition of consensus | Methods Section 2.2.2 |
| **16. Justification of consensus threshold** | Rationale or citation supporting consensus criteria | Methods Section 2.2.2 |
| **17. Quantitative analysis** | Statistical or scoring methods used | Methods Section 2.2.2 |
| **18. Qualitative methods** | Use of qualitative data to inform consensus | Methods Section 2.2.3 |
| **19. Integration of qualitative findings** | How qualitative findings informed subsequent rounds | Methods Sections 2.2.3 and 2.2.4 |
| **20. Management of non-consensus** | Handling of items not reaching consensus | Methods Section 2.2.4 |
| **21. Attrition reporting** | Reporting of participant retention across rounds | Results |
| **22. Ethical considerations** | Informed consent and ethical approval | Methods Section 2.2.1 |
| **23. Methodological limitations** | Acknowledgement of Delphi-related limitations | Discussion |
| **24. Transparency and reproducibility** | Sufficient methodological detail for replication | Methods Section 2.2 |
| **25. Reporting standard adherence** | Explicit alignment with CREDES guidance | Supplementary File B(this checklist) |

Reference:

Jünger, S., Payne, S.A., Brine, J., Radbruch, L. and Brearley, S.G., 2017. Guidance on Conducting and REporting DElphi Studies (CREDES) in palliative care: Recommendations based on a methodological systematic review. Palliative medicine, 31(8), pp.684-706.
