# Supplementary material for: Transforming retraining: Supporting racehorses to have successful second careers
Source: PLoS One. 2026 Aug 19;21(8):e0355509. doi: 10.1371/journal.pone.0355509 (PMC13489413; doi:10.1371/journal.pone.0355509)
Supplement: Supplementary file 3 [file pone.0355509.s003.docx]

S2 Delphi Stage 1 Survey: Transition from racehorse to second careers

Introduction

We would like your help as an expert professional retrainer of former racehorses to find out more about retraining methods and practices in the UK, investigating the common features and key stages of successful early retraining. Our aim is to develop a consensus for good practice and to describe common retraining practices including the identification of physical and behavioral characteristics used to monitor progress across the retraining period. Thank you for your support of this project.

This process is likely to include 3 rounds of surveys, one every 2-3 weeks across October and November. To be successful, it is important that your opinion is captured and shapes the content to make this an interactive and iterative process. This initial Stage 1 questionnaire should not take more than 10 minutes to complete.

Please note that all information which is collected about you during the course of the research will be kept securely. The survey is anonymous and collects no identifiable personal data about you, so that you cannot be recognised from it; however, as we will be contacting the same group of people who are taking part in this project, we will keep a record of who has replied. None of your answers will be linked to your details.

It is our intention to present the results within a final consensus statement, which will be presented at a relevant conference, published in an appropriate journal, and disseminated to a wider audience via articles in the equestrian lay press, as well as being shared with the Hong Kong Jockey Club, as the funders of this project.

This survey is a Hartpury University and Hong Kong Jockey Club research project (ethics number: ETHICS2022-14 ). The research team comprises: Dr Jane Williams, Dr Gillian Tabor, Dr Kathryn Nankervis, Laura Friend, Saranna Jordan, Jess Williams and Steph Evans.  

 If you have any questions or comments regarding this survey please use the box provided at the end of the survey or email Dr Gillian Tabor at gillian.tabor@hartpury.ac.uk
Thank you again for supporting our work.

Consent

It is up to you to decide whether or not to take part in this research. By filling out this questionnaire you are indicating your voluntary informed consent for your results to be included in data analysis. You are free to withdraw without giving a reason up the point of pressing submit at the end of the survey. I acknowledge that my participation in the study is voluntary, I am over 18 years of age, and that I provide consent for the data provided to be used for research purposes.

- Yes I provide consent

Q1 Please can you tick which role best describes your involvement in retraining:

- Trainer
- Veterinarian
- Musculoskeletal therapist

Q2 How many years have you / your facility / your team been involved with retraining former racehorses?

- Less than 1 year
- 1 to 3 years
- 3 to 5 years
- 5 to 10 years
- Longer than 10 years

Q3 How many former racehorses (approximately) have you retrained / been involved with retraining?

- 1 to 5 racehorses
- 6 to 10 racehorses
- 11 to 15 racehorses
- 16 to 20 racehorses
- 20 to 50 racehorses
- More than 50 racehorses

Q4 Based on your experience, please rate how influential you feel the following areas are within a successful racehorse retraining programme? Please select the answer which most closely represents your view:

|  | Always influential (1) | Important but not always influential (2) | Not influential (3) |
| --- | --- | --- | --- |
| Amount of exercise (1) |  |  |  |
| Amount of training (2) |  |  |  |
| Type of exercise (3) |  |  |  |
| Different posture (e.g., how the horse holds itself) (4) |  |  |  |
| Change in the horse's shape (e.g., musculature) (5) |  |  |  |
| Change of daily routine from racing (6) |  |  |  |
| Change of feeding forage and feeding concentrates (7) |  |  |  |
| Change of turnout (8) |  |  |  |
| Amount of free exercise (not ridden or part of training / exercise programme) (9) |  |  |  |
| The rider/s (10) |  |  |  |
| The handler/s (11) |  |  |  |
| Type of training: flat, national hunt or unraced (12) |  |  |  |
| Horse's temperament (13) |  |  |  |

Q5 Are there any other physical or behavioural factors which you feel influence the success of a racehorse's retraining programme? Please describe these below.

________________________________________________________________

Q6 How do you measure/monitor behavioural factors during retraining? Examples of these factors are horses’ demeanour, anxiety levels, ability to cope mentally with work and their trainability. Please describe below.

________________________________________________________________

Q7 Based on your experience, please rate how influential you feel the following areas related to horse health and injury are within a successful racehorse retraining programme? Please select the answer which most closely represents your view:

|  | Always influential (1) | Important but not always influential (2) | Not influential (3) |
| --- | --- | --- | --- |
| Muscle issues (1) |  |  |  |
| Skeletal back pain (2) |  |  |  |
| Pelvic \| Sacro-iliac (SI) \| Thoraco-lumbar (TL) pain (3) |  |  |  |
| Persistent lameness (4) |  |  |  |
| Intermittent lameness (5) |  |  |  |

Q8 Are there any other health or injury related factors which you feel influence the success of a racehorse's retraining programme? Please describe these below.

________________________________________________________________

Q9 Based on your experience, please rate how essential you feel the following activities are within a successful racehorse retraining programme? Please select the answer which most closely represents your view:

|  | Always essential | Important but not essential | Not essential |
| --- | --- | --- | --- |
| Pole work |  |  |  |
| Grid work |  |  |  |
| In-hand / ground work |  |  |  |
| Hacking (alone) |  |  |  |
| Hacking (in company) ( |  |  |  |
| Basic flatwork e.g., simple transitions |  |  |  |
| Advanced flatwork e.g., lateral work |  |  |  |
| Work in open spaces |  |  |  |
| Work on varied terrain |  |  |  |
| Work on the lunge |  |  |  |

Q10 Are there any other activities which you feel are essential to include in a successful racehorse retraining programme? Please describe these below.

________________________________________________________________

Q11 For the activities listed below, approximately how many weeks after the start of the retraining process would you introduce them within your retraining plan? Please insert as whole weeks, e.g., if you would introduce hacking 2 weeks after the start of retraining, you would insert '2'.

- Pole work	__________________________________________________
- Grid work	__________________________________________________
- In hand / ground work _______________________________________
- Hacking (alone) _____________________________________________
- Hacking (in company) ________________________________________
- Basic flatwork e.g., simple transitions ___________________________
- Advanced flatwork e.g., lateral work ___________________________
- Work in open spaces _________________________________________
- Work on varied terrain _______________________________________

Q12 What is the average amount of time you would expect to have a former racehorse in your care for retraining for? Please write your answer below as a whole number and state if this is weeks, months or years e.g., 4 weeks.

________________________________________________________________

Q13 How often would you use the following factors to assess a racehorse’s progress during the retraining process?

|  | Never | Daily | Weekly | Monthly | Every 2 months | Every 6 months | Annually |
| --- | --- | --- | --- | --- | --- | --- | --- |
| Body condition score |  |  |  |  |  |  |  |
| Level of soundness |  |  |  |  |  |  |  |
| Monitoring of previous injury |  |  |  |  |  |  |  |
| Progress in jumping |  |  |  |  |  |  |  |
| Progress in flatwork |  |  |  |  |  |  |  |
| Muscle size / profile |  |  |  |  |  |  |  |
| Palpation: feeling through/over muscles and/or lower limb |  |  |  |  |  |  |  |
| Respiratory rate at rest or exercise |  |  |  |  |  |  |  |
| Heart rate at rest or exercise |  |  |  |  |  |  |  |
| Thoracic back profile (top line profile) |  |  |  |  |  |  |  |

Q14 How do you know when a former racehorse is successfully retrained? Please describe below.

________________________________________________________________

Q15 What would make you change from an expected/standard retraining plan for a former racehorse? Please describe below.

________________________________________________________________

Q16 What are the main challenges you face as a retrainer of racehorses? Please describe below.

________________________________________________________________

Q17 Reflecting on your experience, are there any other factors that you feel impact the success of former racehorses in retraining or within their future careers? Please describe below.

________________________________________________________________

Comments If you would like to add any further comments on retraining racehorses or the Stage 1 survey, please include these below.

________________________________________________________________
