# Supplementary material for: Transforming retraining: Supporting racehorses to have successful second careers
Source: PLoS One. 2026 Aug 19;21(8):e0355509. doi: 10.1371/journal.pone.0355509 (PMC13489413; doi:10.1371/journal.pone.0355509)
Supplement: Supplementary file 4 [file pone.0355509.s004.docx]

**S4 Table S1:** **Frequency of factors which respondents use when selecting former racehorses for retraining.**

|  | Always  (%) | Very likely  (%) | Likely  (%) | Unlikely  (%) | Very unlikely  (%) | Never  (%) |
| --- | --- | --- | --- | --- | --- | --- |
| Intuition | 41 | 21 | 25 | 9 | 2 | 2 |
| Cost | 19 | 21 | 40 | 18 | 0 | 2 |
| Emotional attachment | 11 | 11 | 38 | 30 | 9 | 2 |
| Assessment tool /system | 4 | 4 | 20 | 35 | 16 | 22 |
| Conformation | 30 | 32 | 34 | 4 | 0 | 0 |
| Prior health history | 30 | 33 | 35 | 2 | 0 | 0 |
| Physical assessment | 39 | 39 | 21 | 2 | 0 | 0 |
| Behaviour assessment | 34 | 41 | 16 | 7 | 0 | 2 |
| Temperament assessment | 46 | 33 | 19 | 2 | 0 | 0 |
| Breeding | 2 | 7 | 12 | 47 | 18 | 14 |
