# Supplementary material for: Transforming retraining: Supporting racehorses to have successful second careers
Source: PLoS One. 2026 Aug 19;21(8):e0355509. doi: 10.1371/journal.pone.0355509 (PMC13489413; doi:10.1371/journal.pone.0355509)
Supplement: Supplementary file 5 [file pone.0355509.s005.docx]

**S5 Table S2:** **Frequency of factors which influence how long respondents will retrain a former racehorse for.**

|  | Always  (%) | Very likely  (%) | Likely  (%) | Unlikely  (%) | Very unlikely  (%) | Never  (%) |
| --- | --- | --- | --- | --- | --- | --- |
| Physical health | 70 | 21 | 9 | 0 | 0 | 0 |
| Prior injuries | 64 | 18 | 16 | 2 | 0 | 0 |
| Mental wellbeing | 68 | 21 | 9 | 2 | 0 | 0 |
| Suitability for selected discipline | 64 | 18 | 16 | 2 | 0 | 0 |
| Trainability | 64 | 27 | 9 | 0 | 0 | 0 |
| Affordability | 30 | 18 | 21 | 23 | 4 | 5 |
| Time constraints | 19 | 19 | 37 | 18 | 0 | 7 |
| Suitability of premises / livery | 25 | 23 | 38 | 5 | 4 | 5 |
