# Supplementary material for: Transforming retraining: Supporting racehorses to have successful second careers
Source: PLoS One. 2026 Aug 19;21(8):e0355509. doi: 10.1371/journal.pone.0355509 (PMC13489413; doi:10.1371/journal.pone.0355509)
Supplement: Supplementary file 6 [file pone.0355509.s006.docx]

**S6 Table S3**: **Respondent rating of importance of factors when deciding which discipline or activity a former racehorse is suited for.**

|  | Not at all important  (%) | Slightly important  (%) | Moderately important  (%) | Very important  (%) | Extremely important  (%) |
| --- | --- | --- | --- | --- | --- |
| Conformation | 4 | 16 | 28 | 42 | 11 |
| Stable manners | 3 | 28 | 38 | 17 | 14 |
| Interaction with humans | 5 | 14 | 36 | 31 | 14 |
| New rider's / owner' experience | 2 | 7 | 5 | 38 | 48 |
| Temperament / personality | 0 | 7 | 10 | 47 | 36 |
| Rideability | 2 | 3 | 19 | 45 | 31 |
| Trainability | 2 | 3 | 14 | 55 | 26 |
| Athleticism | 3 | 3 | 41 | 31 | 21 |
| Level of training | 21 | 28 | 29 | 10 | 12 |
| Health | 2 | 2 | 12 | 52 | 33 |
| Able to be ridden alone | 3 | 14 | 34 | 34 | 14 |
| Able to be ridden in company | 3 | 12 | 31 | 36 | 17 |
