# Supplementary material for: Transforming retraining: Supporting racehorses to have successful second careers
Source: PLoS One. 2026 Aug 19;21(8):e0355509. doi: 10.1371/journal.pone.0355509 (PMC13489413; doi:10.1371/journal.pone.0355509)
Supplement: Supplementary file 7 [file pone.0355509.s007.docx]

**Supplementary Information File E: Figure S1: Key challenges respondents identify when rehoming former racehorses.**

| **Horse** | *Attitude* | - Teaching to relax - Reducing anxiety - Reducing flight response - Training challenges e.g., - mounting block |
| --- | --- | --- |
|  | *Developing for a new career* | - Developing strength for new ‘job’ - Topline / muscle profile - Changing posture - Unlearn / learn – retraining not just training, need clarity in approach (fast learners of good / bad) - Soundness - Managing existing conditions - Initial change post exit from racing (can be different horse to what bought) |
| **Perception** | *Appreciation that process is individual (to the horse)* | - Economic cost - Retraining takes time |
|  | *Public perception* | - Lack of understanding of process - Lack of understanding of thoroughbreds/horses |
|  | *People* | - Lack of suitability / experience for a former racehorse |
