# Supplementary material for: Transforming retraining: Supporting racehorses to have successful second careers
Source: PLoS One. 2026 Aug 19;21(8):e0355509. doi: 10.1371/journal.pone.0355509 (PMC13489413; doi:10.1371/journal.pone.0355509)
Supplement: Supplementary file 8 [file pone.0355509.s008.docx]

**Supplementary File D: Table S4: Delphi Round 1 expert consensus overview.**

| **TRAINING** | **CVR** |
| --- | --- |
| Amount of exercise | -0.5 |
| Rider/s | 0.5 |
| Handler/s | 0.7 |
| Type of training | -0.5 |
| Horse's temperament | -0.5 |
| Amount of training | 0.1 |
| Type of exercise | 0.4 |
| Different posture (how the horse holds itself) | 0.4 |
| Changes in the horse’s shape (musculature) | 0.5 |
| Changes in horse's daily routine | -0.1 |
| Change in feeding: forage and/or concentrates | 0.6 |
| Changes in turnout | 0.2 |
| Amount of free exercise (not ridden or part of a training regime) | 0.1 |
| **HORSE HEALTH AND INJURY** | **CVR** |
| Muscle issues | 0.1 |
| Skeletal back pain | 0.5 |
| Pelvic, Sacro-iliac (SI), Thoracolumbar (TL) pain | 0.2 |
| Persistent lameness | 1.0 |
| Intermittent lameness | 0.8 |
| **ACTIVITIES DURING REHABILITATION** | **CVR** |
| Polework | 0.2 |
| Lunge | -0.2 |
| Gridwork | -0.7 |
| In hand / groundwork | 0.1 |
| Hacking alone | 0.6 |
| Hacking in company (other horses) | 0.6 |
| Basic flatwork e.g. simple transitions between gaits | 1.0 |
| Advanced flatwork e.g. lateral work | 0.5 |
| Working in open spaces e.g. field | -0.1 |
| Work on varied terrain e.g. up and downhill | -0.2 |
| **ADDITIONAL AREAS PROPOSED FOR INCLUSION IN ROUND 2 FROM ROUND 1 OPEN QUESTIONS** | |
| Additional information to refine factor definitions was suggested and incorporated into Phase 2 including replacing persistent and intermittent lameness review to assessment of soundness and replacing specific sources of pain e.g. muscle issues, skeletal and pelvic, SI and TL with a more overarching. | |
| **ADDITIONAL AREAS PROPOSED FOR INCLUSION IN ROUND 2 FROM INTERMEDIATE FOCUS GROUPS** | |
| Evaluation of a horse’s posture and muscle profile was considered important. Phrasing was adapted and these areas were included in Round 2.  More overarching monitoring concepts were suggested which were assessed using likert scales to report percentage agreement for different daily and weekly monitoring areas flagged as potential considerations e.g. injury, soundness, musculoskeletal and training progression. | |

Content validity ratios (CVR) were calculated for all factors considered by the Delphi panel experts as essential to be included in the transition period for former racehorses from racing to successful second careers. Factors with a CVR above 0.5 / below -0.5 in Round 1 were retained for further consideration in Round 2.
