# Supplementary material for: Transforming retraining: Supporting racehorses to have successful second careers
Source: PLoS One. 2026 Aug 19;21(8):e0355509. doi: 10.1371/journal.pone.0355509 (PMC13489413; doi:10.1371/journal.pone.0355509)
Supplement: Supplementary file 9 [file pone.0355509.s009.docx]

**Supplementary File I: Table S5: Delphi Round 2 expert consensus overview.**

| **Factors essential to a successful transition for former racehorses** | **CVR** |
| --- | --- |
| Amount of exercise | -0.5 |
| Effect of rider/s on horse’s progress | 1.0 |
| Effect /impact of handler/s on horse’s progress | 1.0 |
| Type of training | -0.5 |
| Horse's temperament | -1.0 |
| Type of exercise undertaken across retraining | 0.8 |
| Evaluating posture or how the horse holds itself to measure progress | 0.8 |
| Changes in the horse’s shape (musculature) and how these have responded to exercise / training | 0.8 |
| Change in feeding: forage and/or concentrates | 1.0 |
| Changes in daily turnout: frequency and type | 0.8 |
| **Essential components within transition programmes** | **CVR** |
| Hacking in company (other horses) | 0.8 |
| Basic flatwork e.g. simple transitions between gaits | 1.0 |
| In hand / groundwork undertaken by experienced handlers to support muscle development | 0.5 |
| Hacking alone | 0.5 |
| Working horse on varied terrain e.g. different surfaces integrating work up and down hill | 0.7 |

From the 28 factors considered in Stage 1 of the Delphi, 14 were retained with phrasing amended as listed. Content validity ratios (CVR) were calculated for all factors considered by the Delphi panel experts as essential to be included in the transition period for former racehorses from racing to successful second careers. Factors with a CVR above 0.7 in Round 2 achieved consensus they should be integrated into transition (indicated by green shading).
