# Supplementary material for: Transforming retraining: Supporting racehorses to have successful second careers
Source: PLoS One. 2026 Aug 19;21(8):e0355509. doi: 10.1371/journal.pone.0355509 (PMC13489413; doi:10.1371/journal.pone.0355509)
Supplement: Supplementary file 10 [file pone.0355509.s011.docx]

PLOS ONE Decision: Revision required [PONE-D-26-00077] - [EMID:17289df17aa76a98]

**Response to Reviewers**

Please include the following items when submitting your revised manuscript:

- A letter that responds to each point raised by the academic editor and reviewer(s). You should upload this letter as a separate file labelled 'Response to Reviewers'.
- A marked-up copy of your manuscript that highlights changes made to the original version. You should upload this as a separate file labelled 'Revised Manuscript with Track Changes'.
- An unmarked version of your revised paper without tracked changes. You should upload this as a separate file labelled 'Manuscript'.

These have been included in our resubmission.

When submitting your revision, we need you to address these additional requirements.

1. Please ensure that your manuscript meets PLOS ONE's style requirements, including those for file naming. The PLOS ONE style templates can be found at

[https://journals.plos.org/plosone/s/file?id=wjVg/PLOSOne_formatting_sample_main_body.pdf](https://linkprotect.cudasvc.com/url?a=https%3a%2f%2ftrack.editorialmanager.com%2fCL0%2fhttps%3a%252F%252Fjournals.plos.org%252Fplosone%252Fs%252Ffile%253Fid%3dwjVg%252FPLOSOne_formatting_sample_main_body.pdf%2f1%2f010f019cff908051-0b8e9255-70c5-4ff9-9b22-c36efa64671b-000000%2f0gritQWf4qX50kWg8xsYtk4CGjdcK2qRuaj-PzP4n8I%3d255&c=E,1,dlwpWMnZoMZapBEUy1xVWhor8ZbVOwmVx2hjQLx-sVw0KCajhhWLcKq0hR0owU4EEY0J_tygrbH_wV5-G1sszaE9RLIQM86g8IyOssaDw5okE8LnitHEfToH&typo=1) and [https://journals.plos.org/plosone/s/file?id=ba62/PLOSOne_formatting_sample_title_authors_affiliations.pdf](https://linkprotect.cudasvc.com/url?a=https%3a%2f%2ftrack.editorialmanager.com%2fCL0%2fhttps%3a%252F%252Fjournals.plos.org%252Fplosone%252Fs%252Ffile%253Fid%3dba62%252FPLOSOne_formatting_sample_title_authors_affiliations.pdf%2f1%2f010f019cff908051-0b8e9255-70c5-4ff9-9b22-c36efa64671b-000000%2f4ebs-DoUvgkkLFXHWX0ij2DG6dNqH0xBzi3dZ-9sZO4%3d255&c=E,1,iTpB6ElmhwGq6dci0wMt6QfForD9YpmibTfp3FNFpH-B6kFxeyU2P2UUQSYPXb46K6hTj0TsVD5KOiARdccRCqRHqF5ZqcOp8gi2zh2L&typo=1)

We can confirm that we have checked the manuscript and associated files to ensure these align to PlosOne style templates.

2. Thank you for stating the following financial disclosure:

[Hong Kong Jockey Club Equine Welfare Research Foundation, grant number PPF-2022-100033].

Please state what role the funders took in the study.  If the funders had no role, please state: "The funders had no role in study design, data collection and analysis, decision to publish, or preparation of the manuscript."

If this statement is not correct you must amend it as needed.

Please include this amended Role of Funder statement in your cover letter; we will change the online submission form on your behalf.

We can confirm that the funding information is correct and that the below statement accurately reflects the role of the funders in publication:

*This study was funded by the Hong Kong Jockey Club Equine Welfare Research Foundation, grant number PPF-2022-100033. The funders had no role in study design, data collection and analysis, decision to publish, or preparation of the manuscript.*

3. Please note that funding information should not appear in any section or other areas of your manuscript. We will only publish funding information present in the Funding Statement section of the online submission form. Please remove any funding-related text from the manuscript.

We can confirm that funder information was within the draft manuscript in the Funding Statement section, which we have now removed.

4. Thank you for stating the following in the Competing Interests section:

[I have read the journal's policy and the authors of this manuscript have the following competing interests: J Cheung is employed as Retraining Manager with HK Jockey Club].

Please confirm that this does not alter your adherence to all PLOS ONE policies on sharing data and materials, by including the following statement: "This does not alter our adherence to PLOS ONE policies on sharing data and materials.” (as detailed online in our guide for authors [http://journals.plos.org/plosone/s/competing-interests](https://linkprotect.cudasvc.com/url?a=http%3a%2f%2fjournals.plos.org%2fplosone%2fs%2fcompeting-interests&c=E,1,aKo40cUvP0zS1o-1jCR7y80dYGe_p37S1Y002Ie9yvbwGyQoP0qVsKmHf5TiiK6Vk6tdmOi-mv9F6_0aZpgnzZw7re6hIcyWVm4nxd0A0pxR6yUEyT3BUVIG&typo=1&ancr_add=1)).  If there are restrictions on sharing of data and/or materials, please state these. Please note that we cannot proceed with consideration of your article until this information has been declared.

Please include your updated Competing Interests statement in your cover letter; we will change the online submission form on your behalf.

We can confirm that while Jessica Cheung is employed by the Hong Kong Jockey Club this does not alter adherence to PLOS ONE policies on sharing data and materials, and have updated our statement to the below:

*The authors declare no conflicts of interest. J.C. is employed as Retired Horse Welfare Manager by the Hong Kong Jockey Club. This does not alter our adherence to PLOS ONE policies on sharing data and materials. The funders had no role in the design of the study; in the collection, analyses, or interpretation of data; in the writing of the manuscript; or in the decision to publish the results.*

5. When completing the data availability statement of the submission form, you indicated that you will make your data available on acceptance. We strongly recommend all authors decide on a data sharing plan before acceptance, as the process can be lengthy and hold up publication timelines. Please note that, though access restrictions are acceptable now, your entire data will need to be made freely accessible if your manuscript is accepted for publication. This policy applies to all data except where public deposition would breach compliance with the protocol approved by your research ethics board. If you are unable to adhere to our open data policy, please kindly revise your statement to explain your reasoning and we will seek the editor's input on an exemption. Please be assured that, once you have provided your new statement, the assessment of your exemption will not hold up the peer review process.

Thank you for your guidance on this matter. Our ethical approval supports data sharing for the anonymised survey data which will be available from Hartpury’s online repository but does not support sharing of interview and focus group transcripts. We have amended our data availability statement to the below which hopefully outlines our position:

*All data underlying this study are not publicly available due to ethical and privacy restrictions. Survey data are available from [URL to be entered] however the dataset also contains information that could compromise the privacy of research participants, specifically interview and focus groups transcripts. Interested researchers may request access to these data by contacting the corresponding author (*[*jane.williams@hartpury.ac.uk*](mailto:jane.williams@hartpury.ac.uk)*) Requests will be considered on a case-by-case basis in accordance with ethical guidelines and institutional data sharing policies.*

6. We note you have included a table to which you do not refer in the text of your manuscript. Please ensure that you refer to Table 4 in your text; if accepted, production will need this reference to link the reader to the table.

Our apologies, this should have been referred to and was incorrectly linked to Table 5. We have included reference to Table 4 on Line 418.

7. We note that this manuscript consists of interview transcripts. Can you please confirm that all participants gave consent for interview transcript to be published?

If they DID provide consent for these transcripts to be published, please also confirm that the transcripts do not contain any potentially identifying information (or let us know if the participants consented to having their personal details published and made publicly available). We consider the following details to be identifying information:

- Names, nicknames, and initials
- Age more specific than round numbers
- GPS coordinates, physical addresses, IP addresses, email addresses
- Information in small sample sizes (e.g. 40 students from X class in X year at X university)
- Specific dates (e.g. visit dates, interview dates)
- ID numbers

Or, if the participants DID NOT provide consent for these transcripts to be published:

- Provide a de-identified version of the data or excerpts of interview responses
- Provide information regarding how these transcripts can be accessed by researchers who meet the criteria for access to confidential data, including:

a) the grounds for restriction
b) the name of the ethics committee, Institutional Review Board, or third-party organization that is imposing sharing restrictions on the data
c) a non-author, institutional point of contact that is able to field data access queries, in the interest of maintaining long-term data accessibility.
d) Any relevant data set names, URLs, DOIs, etc. that an independent researcher would need in order to request your minimal data set.

For further information on sharing data that contains sensitive participant information, please see: [https://journals.plos.org/plosone/s/data-availability#loc-human-research-participant-data-and-other-sensitive-data](https://linkprotect.cudasvc.com/url?a=https%3a%2f%2fjournals.plos.org%2fplosone%2fs%2fdata-availability%23loc-human-research-participant-data-and-other-sensitive-data&c=E,1,RP6jCuTae7nkLjE3Aa7vybsUYI6ouSGo6L1tgvLacMdGE06ilIrh_P1bE9bPBpq0TB3vtiQUP147k3Ui3pyQzAyPaMGllsv_3inJtwWFjF50v4qj&typo=1&ancr_add=1)

If there are ethical, legal, or third-party restrictions upon your dataset, you must provide all of the following details ([https://journals.plos.org/plosone/s/data-availability#loc-acceptable-data-access-restrictions](https://linkprotect.cudasvc.com/url?a=https%3a%2f%2fjournals.plos.org%2fplosone%2fs%2fdata-availability%23loc-acceptable-data-access-restrictions&c=E,1,yj3-VVnpGuus4khfMoZVD0jhuOZ_PgyGZigg5p5Hvod8sEE0wyUApfSypDSIuz2MIg6fJDySd2sr5-MGhutYVF4FwCo-vnG666zZ7QGqOkNVc3LyFIZ_&typo=1&ancr_add=1)):

1. A complete description of the dataset
2. The nature of the restrictions upon the data (ethical, legal, or owned by a third party) and the reasoning behind them
3. The full name of the body imposing the restrictions upon your dataset (ethics committee, institution, data access committee, etc)
4. If the data are owned by a third party, confirmation of whether the authors received any special privileges in accessing the data that other researchers would not have
5. Direct, non-author contact information (preferably email) for the body imposing the restrictions upon the data, to which data access requests can be sent

If the reviewer comments include a recommendation to cite specific previously published works, please review and evaluate these publications to determine whether they are relevant and should be cited. There is no requirement to cite these works unless the editor has indicated otherwise. 

Thank you for highlighting the presence of interview transcripts within this dataset and guidance in this matter. Our ethical approval allows data sharing of anonymised data but not full transcripts, therefore I can confirm on behalf of the authors that the data availability statement should be updated to the below:

*All data underlying this study are not publicly available due to ethical and privacy restrictions. Survey data are available from [URL to be entered] however the dataset also contains information that could compromise the privacy of research participants, specifically interview and focus groups transcripts. Interested researchers may request access to these data by contacting the corresponding author (*[*jane.williams@hartpury.ac.uk*](mailto:jane.williams@hartpury.ac.uk)*) Requests will be considered on a case-by-case basis in accordance with ethical guidelines and institutional data sharing policies.*

**Reviewers' comments: Reviewer's Responses to Questions**

**Comments to the Author**

1. Is the manuscript technically sound, and do the data support the conclusions?

The manuscript must describe a technically sound piece of scientific research with data that supports the conclusions. Experiments must have been conducted rigorously, with appropriate controls, replication, and sample sizes. The conclusions must be drawn appropriately based on the data presented.

Reviewer #1: Yes

Reviewer #2: Partly

2. Has the statistical analysis been performed appropriately and rigorously?

Reviewer #1: Yes

Reviewer #2: I Don't Know

3. Have the authors made all data underlying the findings in their manuscript fully available?

The [PLOS Data policy](https://linkprotect.cudasvc.com/url?a=http%3a%2f%2ftrack.editorialmanager.com%2fCL0%2fhttp%3a%252F%252Fwww.plosone.org%252Fstatic%252Fpolicies.action%2523sharing%2f1%2f010f019cff908051-0b8e9255-70c5-4ff9-9b22-c36efa64671b-000000%2f7-o_aNnLNBudc1VTZPRofW1Rh9u2sWUTueGlDOHHCJQ%3d255&c=E,1,aeWXqJMu097frr8lCgroE-nNP_5o5VbisnhgE6B5cO_gRlhCIBXQnJAvj4qFsCBOJbj_I7I_LDRAG_0pg7_mjWnCnkgwoSe6MgHWTtr3KyU8TFeQ2Dbe&typo=1) requires authors to make all data underlying the findings described in their manuscript fully available without restriction, with rare exception (please refer to the Data Availability Statement in the manuscript PDF file). The data should be provided as part of the manuscript or its supporting information, or deposited to a public repository. For example, in addition to summary statistics, the data points behind means, medians and variance measures should be available. If there are restrictions on publicly sharing data—e.g. participant privacy or use of data from a third party—those must be specified.

Reviewer #1: Yes

Reviewer #2: No

4. Is the manuscript presented in an intelligible fashion and written in standard English?

PLOS ONE does not copyedit accepted manuscripts, so the language in submitted articles must be clear, correct, and unambiguous. Any typographical or grammatical errors should be corrected at revision, so please note any specific errors here.

Reviewer #1: Yes

Reviewer #2: Yes

We thank the reviewers for their thoughtful and constructive feedback, which has been invaluable in strengthening the clarity and quality of this revised manuscript.

5. Review Comments to the Author

Reviewer #1: a well written article that has been carefully edited
Some reviewers might find the material too 'common sense' but I believe that it is important information to have out in the refereed base of literature

Thank you for your support with this work, we also feel this is important to publish to support evidence-informed development of the aftercare sector and former racehorse welfare.

Reviewer #2: This manuscript aims to identify factors that influence successful retraining and transition of former racehorses in order to promote positive lifelong outcomes beyond racing. The study combines an online survey with a Delphi consultation involving stakeholders and experienced retrainers to establish consensus across domains relevant to retraining. The topic is timely and highly relevant. The social license to operate within the racehorse industry increasingly depends on demonstrating long-term welfare sustainability for horses after retirement from racing. Therefore, the development of evidence-informed recommendations and guidelines for successful retraining programs is of considerable importance. The inclusion of stakeholder perspectives strengthens the practical relevance of the study. However, several methodological clarifications and improvements in data presentation are necessary before the manuscript can be considered for publication.

Thank you for the support of our work and helpful feedback which we have addressed and hope you will agree improve the revised manuscript submitted.

MAJOR COMMENTS

Materials and Methods – Delphi Consultation (Rounds 1 and 2)

1) Why were only two Delphi rounds conducted? Was consensus achieved according to predefined criteria?

We adopted a modified, iterative Delphi process, this included the two quantitative Delphi rounds which were conducted to determine consensus supported by iterative qualitative consultation via interviews and focus groups which informed the final round survey. Consensus was achieved according to predefined criteria (≥70% agreement) for all retained domains following Round 2. Additional rounds were therefore unnecessary, consistent with recommendations that Delphi studies should continue until stability of responses is achieved rather than adhering to a fixed number of rounds (Hasson et al., 2000; Diamond et al., 2014). We have clarified this rationale in the revised Methods section.

2) Was the intermediate qualitative focus group planned a priori, or did it emerge as a result of findings from Round 1?

The intermediate focus group stage was planned a priori as part of a modified Delphi design. This approach was adopted to allow qualitative exploration and refinement of domains between quantitative rounds, consistent with established mixed-methods Delphi approaches (Keeney et al., 2011). We have amended the text to make this explicit.

3) What was the time interval between each study phase?

We thank the reviewer for highlighting this omission. The Phase 1 survey took place April-June 2023. The Delphi Round 1 was conducted in October 2023, the focus groups took place approximately 8 weeks later, and Round 2 was completed 8 weeks following the focus groups. This information has been added to the revised manuscript.

4) How many experts were invited to participate in the Delphi consultation, and how many accepted? Although three core professional groups are described as targeted, only two are represented in the Results section. Please clarify.

We appreciate this observation. Although veterinary surgeons were targeted for recruitment, no veterinary surgeons who met the eligibility criteria of direct involvement in the retraining of a minimum of 10 former racehorses responded to the invitation to participate. We have clarified this in the revised Methods and Results sections and acknowledged the limited veterinary representation as a study limitation.

5) Were additional eligibility criteria applied?

The primary eligibility criterion was direct involvement in the transition and retraining of a minimum of 10 former racehorses, including contribution to decision-making related to management, training, or rehabilitation. No additional eligibility criteria were applied beyond those described. We have reviewed the Methods section to ensure all criteria are clearly stated.

6) The methods of analysis for both rounds require further clarification. The rationale for the defined critical values should be explicitly justified.

We have expanded the Methods section to clarify the analytical approach. The 70% agreement threshold was selected based on commonly accepted standards in Delphi methodology, where thresholds typically range from 51% to 80% depending on study objectives (Diamond et al., 2014; Jünger et al., 2017). A 70% threshold was considered appropriate for this exploratory study to balance inclusivity with meaningful consensus. The Content Validity Ratio (CVR) was used as a complementary measure to confirm that agreement exceeded chance levels. We have revised the text to justify these choices explicitly. We have also amended the CVR reporting to 1dp to support this approach and clarity for the reader.

7) A brief explanation of the Content Validity Ratio (CVR), including its interpretation and the minimum acceptable value based on panel size, should be provided.

We have expanded the explanation in methods and outlined differences between round 1 and 2 as well as adding additional text to the limitations section to outline how this approach aligns to other studies and literature.

8) The distinction between qualitative and quantitative analyses is currently unclear. It is particularly confusing whether consensus was defined using CVR or percentage agreement. These approaches should be clearly differentiated and justified.

We thank the reviewer for this helpful observation and have restructured the Methods section to clarify the distinction by outlining that:

- Percentage agreement (≥70%) was used as the primary criterion to determine whether consensus had been reached on each domain.
- Content Validity Ratio (CVR) was used as a complementary quantitative measure to confirm that the level of agreement exceeded what would be expected by chance, based on established critical values for the panel size.
- Qualitative analysis of focus group transcripts was conducted thematically to contextualise quantitative findings, identify emerging domains, and refine item wording between rounds.

We have revised the text to present these approaches as distinct but complementary components of the analysis, in line with recommended practice for mixed-methods Delphi studies.

> Materials and Methods – Intermediate Focus Group

The methodological description of the focus group lacks sufficient detail.
1) How many focus group sessions were conducted?

Two were conducted, this is included line 247.

2) Were the five physiotherapists present in both sessions?

Yes, thank you for highlighting we did not include this – now outlined

3) What were the eligibility criteria for participants?

Aged over 18 yrs and alignment to study’s eligibility criteria, provided.

4) Why were all participants from a single professional group? This raises concerns regarding potential selection bias.

Thank you for highlighting this. We recruited via professional networks and this occurred by chance but agree this could suggest selection bias and have added text to the limitations section to address this concern.

5) How was the content analysis performed (inductive vs. deductive approach)?

Apologies on reflection it is clear we did not clarify clearly that we used thematic analysis within the focus groups – we have added text to ensure this is transparent supported by a positionality statement.

6) How did the findings from the focus group inform the development and refinement of Round 2 items?
We have explained the focus groups fed into Round 2 by helping shape content and inform questions by adding: This analysis served to contextualise quantitative findings from Round 1, identify emerging domains, and refine item wording for the Round 2 survey.

Results – Delphi Consultation

1) Demographic characteristics of participants (e.g., profession, years of experience, geographical distribution) should be reported.

Unfortunately we did not collect years of experience per se from the Delphi participants as we ensured they met the inclusion criteria during recruitment and informed consent. All were resident in the UK. We have included this information for clarity.

2) The presentation of results would benefit from a clearer schematic format, such as summary tables indicating item evolution across rounds.

We have amended the results presentation and included a further supplementary file outlining evolution between rounds (Supp File D).

3) There is insufficient information regarding:
a) Acceptance or rejection rates of factors between rounds
b) Modifications made to items
c) The influence of the focus group on item refinement

We thank the reviewer for this observation and have revised the manuscript accordingly.

a) Acceptance or rejection rates: The additional supplementary file D now reports the number of domains meeting consensus thresholds (≥70% agreement; CVR ≥0.70) after each round, and outlines those retained, rejected, or carried forward for re-rating.

b) Modifications made to items: Revisions to item wording between rounds have been documented in the revised Supp File D Table S4.

c) Influence of the focus group: We have clarified that focus group data were analysed thematically to contextualise Round 1 findings, identify emerging domains, and refine item wording for Round 2.

Providing a structured summary of item progression would substantially improve clarity and transparency
Thank you for this feedback, which has hopefully been addressed above through the revisions made.

MINOR COMMENTS

1) If available, it would be helpful to report in the Introduction the mean age at which horses typically leave racing.

Thank you for highlighted, we have added this information.

2) In the survey results section, please review and verify the percentages reported for geographical origin.

Apologies for the confusing presentation, this has been rectified.

3) For improved readability, consider displaying percentage values directly on the bars in Figures 1 and 2.

Thank you for your suggestion, these have been added to Figs 1 and 2.

4) Table 2 title is inconsistent with both the in-text reference and the table content. Consider renaming the actions as “management factors” for consistency.

Thank you for this insight, on reflection we can see physical attributes do not fully describe the areas considered. We have added in the suggested management factors but retained ‘horses’ physical attributes’ as we feel some aspects such as topline are more directly aligned to the latter.

5) The legend of Table 2 should be rephrased to clarify its intended meaning.

We have amended in line with the above which has hopefully made this easier to follow.
