# Supplementary material for: Transforming retraining: Supporting racehorses to have successful second careers
Source: PLoS One. 2026 Aug 19;21(8):e0355509. doi: 10.1371/journal.pone.0355509 (PMC13489413; doi:10.1371/journal.pone.0355509)
Supplement: Supplementary file 11 [file pone.0355509.s012.docx]

PLOS ONE: PONE-D-26-00077 R3

**Response to Reviewer and Editor Comments**

We would like to thank the reviewers and editor for their feedback and time taken to review our manuscript.

Review cited papers / reference list

Complete – 2 citations #11 and #53 have been amended within the manuscript

Additional Editor Comments:

I acknowledge that the previous revision has addressed all reviewer concerns and substantially improved the manuscript. After careful reading of the present submission,

Thank you for your kind words

I have a few additional comments on wording

- The term paraprofessional is problematic in that it is ambiguous (para to what?) and is based on a view that there is a main profession with related professions assisting. Please consider different wordings on lines 102-3 and 502

We appreciate the reviewer's observation regarding the term "paraprofessional." While this terminology is commonly used within equestrian and veterinary industries to describe allied professions such as farriers and musculoskeletal therapists, we recognise that it may lack clarity for readers outside these sectors. To address this, we have revised the wording on lines 102–3 and 502 to use "allied professionals" with examples of who this represents, which hopefully more transparently conveys the relationship of these roles to veterinary practice without implying a hierarchical structure.

- Table 3 “limn” in the first column should probably be limb?

Apologies, this should indeed be limb and has been amended.

- Line 396 I suggest: "Few respondents (19%) utilised equipment or technology in MONITORING the retraining process"

Thank you for your suggestion, this has been amended.

- Line 733 This is not only about different jurisdictions but also (and perhaps even more) about different sociocultural contexts

Thank you for this insight, we recognise this is also the case and have added a sentence to address that sociocultural differences could also influence application to retraining across different regions.

Comments to the Author

1. If the authors have adequately addressed your comments raised in a previous round of review and you feel that this manuscript is now acceptable for publication, you may indicate that here to bypass the “Comments to the Author” section, enter your conflict of interest statement in the “Confidential to Editor” section, and submit your "Accept" recommendation.

Reviewer #2: All comments have been addressed

Sections 2-5: no response from reviewer

6. Review Comments to the Author

Please use the space provided to explain your answers to the questions above. You may also include additional comments for the author, including concerns about dual publication, research ethics, or publication ethics. (Please upload your review as an attachment if it exceeds 20,000 characters)

Reviewer #2: The authors have adequately addressed my comments and have made substantial revisions to improve the clarity, transparency, and methodological rigour of the manuscript. I have no further major concerns. This manuscript addresses an important and timely topic, and the authors are to be commended for their contribution to improving the successful transition and long-term welfare of former racehorses beyond racing. Further research in this field is encouraged to continue advancing evidence-based retraining practices and sustainable outcomes for these animals.

Thank you for you your time in reviewing the revision and kind feedback.
