# Supplementary material for: Transforming retraining: Supporting racehorses to have successful second careers
Source: PLoS One. 2026 Aug 19;21(8):e0355509. doi: 10.1371/journal.pone.0355509 (PMC13489413; doi:10.1371/journal.pone.0355509)
Supplement: Peer review history

# Peer review: Decision Letter 0

**DOI:** 10.1371/journal.pone.0355509.r001

**Authors:** I Anna Olsson

18 Mar 2026

Transforming Retraining: Supporting racehorses to have successful second careers

PLOS One

Dear Dr. Williams,

Thank you for submitting your manuscript to PLOS ONE. After careful consideration, we feel that it has merit but does not fully meet PLOS ONE’s publication criteria as it currently stands. Therefore, we invite you to submit a revised version of the manuscript that addresses the points raised during the review process.

Please submit your revised manuscript by May 02 2026 11:59PM. If you will need more time than this to complete your revisions, please reply to this message or contact the journal office at plosone@plos.org. When you're ready to submit your revision, log on to https://www.editorialmanager.com/pone/ and select the 'Submissions Needing Revision' folder to locate your manuscript file.

Please include the following items when submitting your revised manuscript:

- A letter that responds to each point raised by the academic editor and reviewer(s). You should upload this letter as a separate file labeled 'Response to Reviewers'.
- A marked-up copy of your manuscript that highlights changes made to the original version. You should upload this as a separate file labeled 'Revised Manuscript with Track Changes'.
- An unmarked version of your revised paper without tracked changes. You should upload this as a separate file labeled 'Manuscript'.

If you would like to make changes to your financial disclosure, please include your updated statement in your cover letter. Guidelines for resubmitting your figure files are available below the reviewer comments at the end of this letter.

If applicable, we recommend that you deposit your laboratory protocols in protocols.io to enhance the reproducibility of your results. Protocols.io assigns your protocol its own identifier (DOI) so that it can be cited independently in the future. For instructions see: https://journals.plos.org/plosone/s/submission-guidelines#loc-laboratory-protocols. Additionally, PLOS ONE offers an option for publishing peer-reviewed Lab Protocol articles, which describe protocols hosted on protocols.io. Read more information on sharing protocols at https://plos.org/protocols?utm_medium=editorial-email&utm_source=authorletters&utm_campaign=protocols.

We look forward to receiving your revised manuscript.

Kind regards,

I Anna S Olsson, Ph.D.

Academic Editor

PLOS One

Journal Requirements:

When submitting your revision, we need you to address these additional requirements.

1. Please ensure that your manuscript meets PLOS ONE's style requirements, including those for file naming. The PLOS ONE style templates can be found at

https://journals.plos.org/plosone/s/file?id=wjVg/PLOSOne_formatting_sample_main_body.pdf and https://journals.plos.org/plosone/s/file?id=ba62/PLOSOne_formatting_sample_title_authors_affiliations.pdf

2. Thank you for stating the following financial disclosure:

[Hong Kong Jockey Club Equine Welfare Research Foundation, grant number PPF-2022-100033].

Please state what role the funders took in the study.  If the funders had no role, please state: "The funders had no role in study design, data collection and analysis, decision to publish, or preparation of the manuscript."

If this statement is not correct you must amend it as needed.

Please include this amended Role of Funder statement in your cover letter; we will change the online submission form on your behalf.

3. Please note that funding information should not appear in any section or other areas of your manuscript. We will only publish funding information present in the Funding Statement section of the online submission form. Please remove any funding-related text from the manuscript.

4. Thank you for stating the following in the Competing Interests section:

[I have read the journal's policy and the authors of this manuscript have the following competing interests: J Cheung is employed as Retraining Manager with HK Jockey Club].

Please confirm that this does not alter your adherence to all PLOS ONE policies on sharing data and materials, by including the following statement: "This does not alter our adherence to PLOS ONE policies on sharing data and materials.” (as detailed online in our guide for authors http://journals.plos.org/plosone/s/competing-interests).  If there are restrictions on sharing of data and/or materials, please state these. Please note that we cannot proceed with consideration of your article until this information has been declared.

Please include your updated Competing Interests statement in your cover letter; we will change the online submission form on your behalf.

5. When completing the data availability statement of the submission form, you indicated that you will make your data available on acceptance. We strongly recommend all authors decide on a data sharing plan before acceptance, as the process can be lengthy and hold up publication timelines. Please note that, though access restrictions are acceptable now, your entire data will need to be made freely accessible if your manuscript is accepted for publication. This policy applies to all data except where public deposition would breach compliance with the protocol approved by your research ethics board. If you are unable to adhere to our open data policy, please kindly revise your statement to explain your reasoning and we will seek the editor's input on an exemption. Please be assured that, once you have provided your new statement, the assessment of your exemption will not hold up the peer review process.

6. We note you have included a table to which you do not refer in the text of your manuscript. Please ensure that you refer to Table 4 in your text; if accepted, production will need this reference to link the reader to the table.

7. We note that this manuscript consists of interview transcripts. Can you please confirm that all participants gave consent for interview transcript to be published?

If they DID provide consent for these transcripts to be published, please also confirm that the transcripts do not contain any potentially identifying information (or let us know if the participants consented to having their personal details published and made publicly available). We consider the following details to be identifying information:

- Names, nicknames, and initials

- Age more specific than round numbers

- GPS coordinates, physical addresses, IP addresses, email addresses

- Information in small sample sizes (e.g. 40 students from X class in X year at X university)

- Specific dates (e.g. visit dates, interview dates)

- ID numbers

Or, if the participants DID NOT provide consent for these transcripts to be published:

- Provide a de-identified version of the data or excerpts of interview responses

- Provide information regarding how these transcripts can be accessed by researchers who meet the criteria for access to confidential data, including:

a) the grounds for restriction

b) the name of the ethics committee, Institutional Review Board, or third-party organization that is imposing sharing restrictions on the data

c) a non-author, institutional point of contact that is able to field data access queries, in the interest of maintaining long-term data accessibility.

d) Any relevant data set names, URLs, DOIs, etc. that an independent researcher would need in order to request your minimal data set.

For further information on sharing data that contains sensitive participant information, please see: https://journals.plos.org/plosone/s/data-availability#loc-human-research-participant-data-and-other-sensitive-data

If there are ethical, legal, or third-party restrictions upon your dataset, you must provide all of the following details (https://journals.plos.org/plosone/s/data-availability#loc-acceptable-data-access-restrictions):

1. A complete description of the dataset

2. The nature of the restrictions upon the data (ethical, legal, or owned by a third party) and the reasoning behind them

3. The full name of the body imposing the restrictions upon your dataset (ethics committee, institution, data access committee, etc)

4. If the data are owned by a third party, confirmation of whether the authors received any special privileges in accessing the data that other researchers would not have

5. Direct, non-author contact information (preferably email) for the body imposing the restrictions upon the data, to which data access requests can be sent

If the reviewer comments include a recommendation to cite specific previously published works, please review and evaluate these publications to determine whether they are relevant and should be cited. There is no requirement to cite these works unless the editor has indicated otherwise.

[Note: HTML markup is below. Please do not edit.]

Reviewers' comments:

Reviewer's Responses to Questions

**Comments to the Author**

1. Is the manuscript technically sound, and do the data support the conclusions?

Reviewer #1: Yes

Reviewer #2: Partly

**********

2. Has the statistical analysis been performed appropriately and rigorously?

Reviewer #1: Yes

Reviewer #2: I Don't Know

**********

3. Have the authors made all data underlying the findings in their manuscript fully available?

The PLOS Data policy

Reviewer #1: Yes

Reviewer #2: No

**********

4. Is the manuscript presented in an intelligible fashion and written in standard English?

Reviewer #1: Yes

Reviewer #2: Yes

**********

Reviewer #1: a well written article that has been carefully edited

some reviewers might find the material too 'common sense' but I believe that it is important information to have out in the refereed base of ltierature

Reviewer #2: This manuscript aims to identify factors that influence successful retraining and transition of former racehorses in order to promote positive lifelong outcomes beyond racing. The study combines an online survey with a Delphi consultation involving stakeholders and experienced retrainers to establish consensus across domains relevant to retraining.

The topic is timely and highly relevant. The social license to operate within the racehorse industry increasingly depends on demonstrating long-term welfare sustainability for horses after retirement from racing. Therefore, the development of evidence-informed recommendations and guidelines for successful retraining programs is of considerable importance. The inclusion of stakeholder perspectives strengthens the practical relevance of the study.

However, several methodological clarifications and improvements in data presentation are necessary before the manuscript can be considered for publication.

MAJOR COMMENTS

> Materials and Methods – Delphi Consultation (Rounds 1 and 2)

1) Why were only two Delphi rounds conducted? Was consensus achieved according to predefined criteria?

2) Was the intermediate qualitative focus group planned a priori, or did it emerge as a result of findings from Round 1?

3) What was the time interval between each study phase?

4) How many experts were invited to participate in the Delphi consultation, and how many accepted? Although three core professional groups are described as targeted, only two are represented in the Results section. Please clarify.

5) Were additional eligibility criteria applied?

6) The methods of analysis for both rounds require further clarification. The rationale for the defined critical values should be explicitly justified.

7) A brief explanation of the Content Validity Ratio (CVR), including its interpretation and the minimum acceptable value based on panel size, should be provided.

8) The distinction between qualitative and quantitative analyses is currently unclear. It is particularly confusing whether consensus was defined using CVR or percentage agreement. These approaches should be clearly differentiated and justified.

> Materials and Methods – Intermediate Focus Group

The methodological description of the focus group lacks sufficient detail.

1) How many focus group sessions were conducted?

2) Were the five physiotherapists present in both sessions?

3) What were the eligibility criteria for participants?

4) Why were all participants from a single professional group? This raises concerns regarding potential selection bias.

5) How was the content analysis performed (inductive vs. deductive approach)?

6) How did the findings from the focus group inform the development and refinement of Round 2 items?

> Results – Delphi Consultation

1) Demographic characteristics of participants (e.g., profession, years of experience, geographical distribution) should be reported.

2) The presentation of results would benefit from a clearer schematic format, such as summary tables indicating item evolution across rounds.

3) There is insufficient information regarding:

a) Acceptance or rejection rates of factors between rounds

b) Modifications made to items

c) The influence of the focus group on item refinement

Providing a structured summary of item progression would substantially improve clarity and transparency.

MINOR COMMENTS

1) If available, it would be helpful to report in the Introduction the mean age at which horses typically leave racing.

2) In the survey results section, please review and verify the percentages reported for geographical origin.

3) For improved readability, consider displaying percentage values directly on the bars in Figures 1 and 2.

4) Table 2 title is inconsistent with both the in-text reference and the table content. Consider renaming the actions as “management factors” for consistency.

5) The legend of Table 2 should be rephrased to clarify its intended meaning.

**********

what does this mean?). If published, this will include your full peer review and any attached files.

If you choose “no”, your identity will remain anonymous but your review may still be made public.

**Do you want your identity to be public for this peer review?**  For information about this choice, including consent withdrawal, please see our Privacy Policy

Reviewer #1: No

Reviewer #2: No

**********

[NOTE: If reviewer comments were submitted as an attachment file, they will be attached to this email and accessible via the submission site. Please log into your account, locate the manuscript record, and check for the action link "View Attachments". If this link does not appear, there are no attachment files.]

To ensure your figures meet our technical requirements, please review our figure guidelines: https://journals.plos.org/plosone/s/figures

You may also use PLOS’s free figure tool, NAAS, to help you prepare publication quality figures: https://journals.plos.org/plosone/s/figures#loc-tools-for-figure-preparation.

NAAS will assess whether your figures meet our technical requirements by comparing each figure against our figure specifications.

# Author comment: Author response to Decision Letter 1

**DOI:** 10.1371/journal.pone.0355509.r002

26 May 2026

PLOS ONE Decision: Revision required [PONE-D-26-00077] - [EMID:17289df17aa76a98]

Response to Reviewers

Please include the following items when submitting your revised manuscript:

• A letter that responds to each point raised by the academic editor and reviewer(s). You should upload this letter as a separate file labelled 'Response to Reviewers'.

• A marked-up copy of your manuscript that highlights changes made to the original version. You should upload this as a separate file labelled 'Revised Manuscript with Track Changes'.

• An unmarked version of your revised paper without tracked changes. You should upload this as a separate file labelled 'Manuscript'.

These have been included in our resubmission.

When submitting your revision, we need you to address these additional requirements.

1. Please ensure that your manuscript meets PLOS ONE's style requirements, including those for file naming. The PLOS ONE style templates can be found at

https://journals.plos.org/plosone/s/file?id=wjVg/PLOSOne_formatting_sample_main_body.pdf and https://journals.plos.org/plosone/s/file?id=ba62/PLOSOne_formatting_sample_title_authors_affiliations.pdf

We can confirm that we have checked the manuscript and associated files to ensure these align to PlosOne style templates.

2. Thank you for stating the following financial disclosure:

[Hong Kong Jockey Club Equine Welfare Research Foundation, grant number PPF-2022-100033].

Please state what role the funders took in the study. If the funders had no role, please state: "The funders had no role in study design, data collection and analysis, decision to publish, or preparation of the manuscript."

If this statement is not correct you must amend it as needed.

Please include this amended Role of Funder statement in your cover letter; we will change the online submission form on your behalf.

We can confirm that the funding information is correct and that the below statement accurately reflects the role of the funders in publication:

This study was funded by the Hong Kong Jockey Club Equine Welfare Research Foundation, grant number PPF-2022-100033. The funders had no role in study design, data collection and analysis, decision to publish, or preparation of the manuscript.

3. Please note that funding information should not appear in any section or other areas of your manuscript. We will only publish funding information present in the Funding Statement section of the online submission form. Please remove any funding-related text from the manuscript.

We can confirm that funder information was within the draft manuscript in the Funding Statement section, which we have now removed.

4. Thank you for stating the following in the Competing Interests section:

[I have read the journal's policy and the authors of this manuscript have the following competing interests: J Cheung is employed as Retraining Manager with HK Jockey Club].

Please confirm that this does not alter your adherence to all PLOS ONE policies on sharing data and materials, by including the following statement: "This does not alter our adherence to PLOS ONE policies on sharing data and materials.” (as detailed online in our guide for authors http://journals.plos.org/plosone/s/competing-interests). If there are restrictions on sharing of data and/or materials, please state these. Please note that we cannot proceed with consideration of your article until this information has been declared.

Please include your updated Competing Interests statement in your cover letter; we will change the online submission form on your behalf.

We can confirm that while Jessica Cheung is employed by the Hong Kong Jockey Club this does not alter adherence to PLOS ONE policies on sharing data and materials, and have updated our statement to the below:

The authors declare no conflicts of interest. J.C. is employed as Retired Horse Welfare Manager by the Hong Kong Jockey Club. This does not alter our adherence to PLOS ONE policies on sharing data and materials. The funders had no role in the design of the study; in the collection, analyses, or interpretation of data; in the writing of the manuscript; or in the decision to publish the results.

5. When completing the data availability statement of the submission form, you indicated that you will make your data available on acceptance. We strongly recommend all authors decide on a data sharing plan before acceptance, as the process can be lengthy and hold up publication timelines. Please note that, though access restrictions are acceptable now, your entire data will need to be made freely accessible if your manuscript is accepted for publication. This policy applies to all data except where public deposition would breach compliance with the protocol approved by your research ethics board. If you are unable to adhere to our open data policy, please kindly revise your statement to explain your reasoning and we will seek the editor's input on an exemption. Please be assured that, once you have provided your new statement, the assessment of your exemption will not hold up the peer review process.

Thank you for your guidance on this matter. Our ethical approval supports data sharing for the anonymised survey data which will be available from Hartpury’s online repository but does not support sharing of interview and focus group transcripts. We have amended our data availability statement to the below which hopefully outlines our position:

All data underlying this study are not publicly available due to ethical and privacy restrictions. Survey data are available from [URL to be entered] however the dataset also contains information that could compromise the privacy of research participants, specifically interview and focus groups transcripts. Interested researchers may request access to these data by contacting the corresponding author (jane.williams@hartpury.ac.uk) Requests will be considered on a case-by-case basis in accordance with ethical guidelines and institutional data sharing policies.

6. We note you have included a table to which you do not refer in the text of your manuscript. Please ensure that you refer to Table 4 in your text; if accepted, production will need this reference to link the reader to the table.

Our apologies, this should have been referred to and was incorrectly linked to Table 5. We have included reference to Table 4 on Line 418.

7. We note that this manuscript consists of interview transcripts. Can you please confirm that all participants gave consent for interview transcript to be published?

If they DID provide consent for these transcripts to be published, please also confirm that the transcripts do not contain any potentially identifying information (or let us know if the participants consented to having their personal details published and made publicly available). We consider the following details to be identifying information:

- Names, nicknames, and initials

- Age more specific than round numbers

- GPS coordinates, physical addresses, IP addresses, email addresses

- Information in small sample sizes (e.g. 40 students from X class in X year at X university)

- Specific dates (e.g. visit dates, interview dates)

- ID numbers

Or, if the participants DID NOT provide consent for these transcripts to be published:

- Provide a de-identified version of the data or excerpts of interview responses

- Provide information regarding how these transcripts can be accessed by researchers who meet the criteria for access to confidential data, including:

a) the grounds for restriction

b) the name of the ethics committee, Institutional Review Board, or third-party organization that is imposing sharing restrictions on the data

c) a non-author, institutional point of contact that is able to field data access queries, in the interest of maintaining long-term data accessibility.

d) Any relevant data set names, URLs, DOIs, etc. that an independent researcher would need in order to request your minimal data set.

For further information on sharing data that contains sensitive participant information, please see: https://journals.plos.org/plosone/s/data-availability#loc-human-research-participant-data-and-other-sensitive-data

If there are ethical, legal, or third-party restrictions upon your dataset, you must provide all of the following details (https://journals.plos.org/plosone/s/data-availability#loc-acceptable-data-access-restrictions):

1. A complete description of the dataset

2. The nature of the restrictions upon the data (ethical, legal, or owned by a third party) and the reasoning behind them

3. The full name of the body imposing the restrictions upon your dataset (ethics committee, institution, data access committee, etc)

4. If the data are owned by a third party, confirmation of whether the authors received any special privileges in accessing the data that other researchers would not have

5. Direct, non-author contact information (preferably email) for the body imposing the restrictions upon the data, to which data access requests can be sent

If the reviewer comments include a recommendation to cite specific previously published works, please review and evaluate these publications to determine whether they are relevant and should be cited. There is no requirement to cite these works unless the editor has indicated otherwise.

Thank you for highlighting the presence of interview transcripts within this dataset and guidance in this matter. Our ethical approval allows data sharing of anonymised data but not full transcripts, therefore I can confirm on behalf of the authors that the data availability statement should be updated to the below:

All data underlying this study are not publicly available due to ethical and privacy restrictions. Survey data are available from [URL to be entered] however the dataset also contains information that could compromise the privacy of research participants, specifically interview and focus groups transcripts. Interested researchers may request access to these data by contacting the corresponding author (jane.williams@hartpury.ac.uk) Requests will be considered on a case-by-case basis in accordance with ethical guidelines and institutional data sharing policies.

Reviewers' comments: Reviewer's Responses to Questions

Comments to the Author

1. Is the manuscript technically sound, and do the data support the conclusions?

The manuscript must describe a technically sound piece of scientific research with data that supports the conclusions. Experiments must have been conducted rigorously, with appropriate controls, replication, and sample sizes. The conclusions must be drawn appropriately based on the data presented.

Reviewer #1: Yes

Reviewer #2: Partly

________________________________________

2. Has the statistical analysis been performed appropriately and rigorously?

Reviewer #1: Yes

Reviewer #2: I Don't Know

________________________________________

3. Have the authors made all data underlying the findings in their manuscript fully available?

The PLOS Data policy requires authors to make all data underlying the findings described in their manuscript fully available without restriction, with rare exception (please refer to the Data Availability Statement in the manuscript PDF file). The data should be provided as part of the manuscript or its supporting information, or deposited to a public repository. For example, in addition to summary statistics, the data points behind means, medians and variance measures should be available. If there are restrictions on publicly sharing data—e.g. participant privacy or use of data from a third party—those must be specified.

Reviewer #1: Yes

Reviewer #2: No

________________________________________

4. Is the manuscript presented in an intelligible fashion and written in standard English?

PLOS ONE does not copyedit accepted manuscripts, so the language in submitted articles must be clear, correct, and unambiguous. Any typographical or grammatical errors should be corrected at revision, so please note any specific errors here.

Reviewer #1: Yes

Reviewer #2: Yes

________________________________________

We thank the reviewers for their thoughtful and constructive feedback, which has been invaluable in strengthening the clarity and quality of this revised manuscript.

5. Review Comments to the Author

Reviewer #1: a well written article that has been carefully edited

Some reviewers might find the material too 'common sense' but I believe that it is important information to have out in the refereed base of literature

Thank you for your support with this work, we also feel this is important to publish to support evidence-informed development of the aftercare sector and former racehorse welfare.

Reviewer #2: This manuscript aims to identify factors that influence successful retraining and transition of former racehorses in order to promote positive lifelong outcomes beyond racing. The study combines an online survey with a Delphi consultation involving stakeholders and experienced retrainers to establish consensus across domains relevant to retraining. The topic is timely and highly relevant. The social license to operate within the racehorse industry increasingly depends on demonstrating long-term welfare sustainability for horses after retirement from racing. Therefore, the development of evidence-informed recommendations and guidelines for successful retraining programs is of considerable importance. The inclusion of stakeholder perspectives strengthens the practical relevance of the study. However, several methodological clarifications and improvements in data presentation are necessary before the manuscript can be considered for publication.

Thank you for the support of our work and helpful feedback which we have addressed and hope you will agree improve the revised manuscript submitted.

MAJOR COMMENTS

Materials and Methods – Delphi Consultation (Rounds 1 and 2)

1) Why were only two Delphi rounds conducted? Was consensus achieved according to predefined criteria?

We adopted a modified, iterative Delphi process, this included the two quantitative Delphi rounds which were conducted to determine consensus supported by iterative qualitative consultation via interviews and focus groups which informed the final round survey. Consensus was achieved according to predefined criteria (≥70% agreement) for all retained domains following Round 2. Additional rounds were therefore unnecessary, consistent with recommendations that Delphi studies should continue until stability of responses is achieved rather than adhering to a fixed number of rounds (Hasson et al., 2000; Diamond et al., 2014). We have clarified this rationale in the revised Methods section.

2) Was the intermediate qualitative focus group planned a priori, or did it emerge as a result of findings from Round 1?

The intermediate focus group stage was planned a priori as part of a modified Delphi design. This approach was adopted to allow qualitative exploration and refinement of domains between quantitative rounds, consistent with established mixed-methods Delphi approaches (Keeney et al., 2011). We have amended the text to make this explicit.

3) What was the time interval between each study phase?

We thank the reviewer for highlighting this omission. The Phase 1 survey took place April-June 2023. The Delphi Round 1 was conducted in October 2023, the focus groups took place approximately 8 weeks later, and Round 2 was completed 8 weeks following the focus groups. This information has been added to the revised manuscript.

4) How many experts were invited to participate in the Delphi consultation, and how many accepted? Although three core professional groups are described as targeted, only two are represented in the Results section. Please clarify.

We appreciate this observation. Although veterinary surgeons were targeted for recruitment, no veterinary surgeons who met the eligibility criteria of direct involvement in the retraining of a minimum of 10 former racehorses responded to the invitation to participate. We have clarified this in the revised Methods and Results sections and acknowledged the limited veterinary representation as a study limitation.

5) Were additional eligibility criteria applied?

The primary eligibility cri

Attachment Submitted filename: Response to reviewers transition.docx

# Peer review: Decision Letter 1

**DOI:** 10.1371/journal.pone.0355509.r003

**Authors:** I Anna Olsson

13 Jul 2026

Dear Dr. Williams,

Please submit your revised manuscript by Aug 27 2026 11:59PM. If you will need more time than this to complete your revisions, please reply to this message or contact the journal office at plosone@plos.org. When you're ready to submit your revision, log on to https://www.editorialmanager.com/pone/ and select the 'Submissions Needing Revision' folder to locate your manuscript file.

- A letter that responds to each point raised by the academic editor and reviewer(s). You should upload this letter as a separate file labeled 'Response to Reviewers'.
- A marked-up copy of your manuscript that highlights changes made to the original version. You should upload this as a separate file labeled 'Revised Manuscript with Track Changes'.
- An unmarked version of your revised paper without tracked changes. You should upload this as a separate file labeled 'Manuscript'.

If you would like to make changes to your financial disclosure, please include your updated statement in your cover letter. Guidelines for resubmitting your figure files are available below the reviewer comments at the end of this letter.

If applicable, we recommend that you deposit your laboratory protocols in protocols.io to enhance the reproducibility of your results. Protocols.io assigns your protocol its own identifier (DOI) so that it can be cited independently in the future. For instructions see: https://journals.plos.org/plosone/s/submission-guidelines#loc-laboratory-protocols. Additionally, PLOS ONE offers an option for publishing peer-reviewed Lab Protocol articles, which describe protocols hosted on protocols.io. Read more information on sharing protocols at https://plos.org/protocols?utm_medium=editorial-email&utm_source=authorletters&utm_campaign=protocols.

As the corresponding author, your ORCID iD is verified in the submission system and will appear in the published article. PLOS supports the use of ORCID, and we encourage all coauthors to register for an ORCID iD and use it as well. Please encourage your coauthors to verify their ORCID iD within the submission system before final acceptance, as unverified ORCID iDs will not appear in the published article. *Only*  the individual author can complete the verification step; PLOS staff *cannot*  verify ORCID iDs on behalf of authors.

We look forward to receiving your revised manuscript.

Kind regards,

I Anna S Olsson, Ph.D.

Academic Editor

PLOS One

Journal Requirements:

1. If the reviewer comments include a recommendation to cite specific previously published works, please review and evaluate these publications to determine whether they are relevant and should be cited. There is no requirement to cite these works unless the editor has indicated otherwise.

2. Please review your reference list to ensure that it is complete and correct. If you have cited papers that have been retracted, please include the rationale for doing so in the manuscript text, or remove these references and replace them with relevant current references. Any changes to the reference list should be mentioned in the rebuttal letter that accompanies your revised manuscript. If you need to cite a retracted article, indicate the article’s retracted status in the References list and also include a citation and full reference for the retraction notice.

Additional Editor Comments:

I acknowledge that the previous revision has addressed all reviewer concerns and substantially improved the manuscript. After careful reading of the present submission, I have a few additional comments on wording:

- The term paraprofessional is problematic in that it is ambiguous (para to what?) and is based on a view that there is a main profession with related professions assisting. Please consider different wordings on lines 102-3 and 502

- Table 3 “limn” in the first column should probably be limb?

- Line 396 I suggest: "Few respondents (19%) utilised equipment or technology in MONITORING the retraining process"

- Line 733 This is not only about different jurisdictions but also (and perhaps even more) about different sociocultural contexts

[Note: HTML markup is below. Please do not edit.]

Reviewer's Responses to Questions

**Comments to the Author**

Reviewer #2: All comments have been addressed

**********

2. Is the manuscript technically sound, and do the data support the conclusions?

Reviewer #2: (No Response)

**********

3. Has the statistical analysis been performed appropriately and rigorously?

Reviewer #2: (No Response)

**********

4. Have the authors made all data underlying the findings in their manuscript fully available?

The PLOS Data policy

Reviewer #2: (No Response)

**********

5. Is the manuscript presented in an intelligible fashion and written in standard English?

Reviewer #2: (No Response)

**********

Reviewer #2: The authors have adequately addressed my comments and have made substantial revisions to improve the clarity, transparency, and methodological rigour of the manuscript. I have no further major concerns. This manuscript addresses an important and timely topic, and the authors are to be commended for their contribution to improving the successful transition and long-term welfare of former racehorses beyond racing. Further research in this field is encouraged to continue advancing evidence-based retraining practices and sustainable outcomes for these animals.

**********

what does this mean?). If published, this will include your full peer review and any attached files.

If you choose “no”, your identity will remain anonymous but your review may still be made public.

**Do you want your identity to be public for this peer review?**  For information about this choice, including consent withdrawal, please see our Privacy Policy

Reviewer #2: No

**********

[NOTE: If reviewer comments were submitted as an attachment file, they will be attached to this email and accessible via the submission site. Please log into your account, locate the manuscript record, and check for the action link "View Attachments". If this link does not appear, there are no attachment files.]

To ensure your figures meet our technical requirements, please review our figure guidelines: https://journals.plos.org/plosone/s/figures

You may also use PLOS’s free figure tool, NAAS, to help you prepare publication quality figures: https://journals.plos.org/plosone/s/figures#loc-tools-for-figure-preparation.

NAAS will assess whether your figures meet our technical requirements by comparing each figure against our figure specifications.

# Author comment: Author response to Decision Letter 2

**DOI:** 10.1371/journal.pone.0355509.r004

21 Jul 2026

We would like to thank the reviewers and editor for their feedback and time taken to review our manuscript.

Review cited papers / reference list

Complete – 2 citations #11 and #53 have been amended within the manuscript

Additional Editor Comments:

I acknowledge that the previous revision has addressed all reviewer concerns and substantially improved the manuscript. After careful reading of the present submission,

Thank you for your kind words

I have a few additional comments on wording

- The term paraprofessional is problematic in that it is ambiguous (para to what?) and is based on a view that there is a main profession with related professions assisting. Please consider different wordings on lines 102-3 and 502

We appreciate the reviewer's observation regarding the term "paraprofessional." While this terminology is commonly used within equestrian and veterinary industries to describe allied professions such as farriers and musculoskeletal therapists, we recognise that it may lack clarity for readers outside these sectors. To address this, we have revised the wording on lines 102–3 and 502 to use "allied professionals" with examples of who this represents, which hopefully more transparently conveys the relationship of these roles to veterinary practice without implying a hierarchical structure.

- Table 3 “limn” in the first column should probably be limb?

Apologies, this should indeed be limb and has been amended.

- Line 396 I suggest: "Few respondents (19%) utilised equipment or technology in MONITORING the retraining process"

Thank you for your suggestion, this has been amended.

- Line 733 This is not only about different jurisdictions but also (and perhaps even more) about different sociocultural contexts

Thank you for this insight, we recognise this is also the case and have added a sentence to address that sociocultural differences could also influence application to retraining across different regions.

Comments to the Author

1. If the authors have adequately addressed your comments raised in a previous round of review and you feel that this manuscript is now acceptable for publication, you may indicate that here to bypass the “Comments to the Author” section, enter your conflict of interest statement in the “Confidential to Editor” section, and submit your "Accept" recommendation.

Reviewer #2: All comments have been addressed

Sections 2-5: no response from reviewer

6. Review Comments to the Author

Please use the space provided to explain your answers to the questions above. You may also include additional comments for the author, including concerns about dual publication, research ethics, or publication ethics. (Please upload your review as an attachment if it exceeds 20,000 characters)

Reviewer #2: The authors have adequately addressed my comments and have made substantial revisions to improve the clarity, transparency, and methodological rigour of the manuscript. I have no further major concerns. This manuscript addresses an important and timely topic, and the authors are to be commended for their contribution to improving the successful transition and long-term welfare of former racehorses beyond racing. Further research in this field is encouraged to continue advancing evidence-based retraining practices and sustainable outcomes for these animals.

Thank you for you your time in reviewing the revision and kind feedback.

Attachment Submitted filename: Plos One Trans R3 response to reviewer and editor comments.docx

# Editor report: Decision Letter 2

**DOI:** 10.1371/journal.pone.0355509.r005

**Authors:** I Anna Olsson

22 Jul 2026

Transforming Retraining: Supporting racehorses to have successful second careers

PONE-D-26-00077R2

Dear Dr. Williams,

We’re pleased to inform you that your manuscript has been judged scientifically suitable for publication and will be formally accepted for publication once it meets all outstanding technical requirements.

Within one week, you’ll receive an e-mail detailing the required amendments. When these have been addressed, you’ll receive a formal acceptance letter and your manuscript will be scheduled for publication.

An invoice will be generated when your article is formally accepted. Please note, if your institution has a publishing partnership with PLOS and your article meets the relevant criteria, all or part of your publication costs will be covered. Please make sure your user information is up-to-date by logging into Editorial Manager at Editorial Manager® and clicking the ‘Update My Information' link at the top of the page. For questions related to billing, please contact billing support.

If your institution or institutions have a press office, please notify them about your upcoming paper to help maximize its impact. If they’ll be preparing press materials, please inform our press team as soon as possible -- no later than 48 hours after receiving the formal acceptance. Your manuscript will remain under strict press embargo until 2 pm Eastern Time on the date of publication. For more information, please contact onepress@plos.org.

Kind regards,

I Anna S Olsson, Ph.D.

Academic Editor

PLOS One

Additional Editor Comments (optional):

Reviewers' comments:

# Editor report: Acceptance letter

**DOI:** 10.1371/journal.pone.0355509.r006

**Authors:** I Anna Olsson

PONE-D-26-00077R2

PLOS One

Dear Dr. Williams,

I'm pleased to inform you that your manuscript has been deemed suitable for publication in PLOS One. Congratulations! Your manuscript is now being handed over to our production team.

At this stage, our production department will prepare your paper for publication. This includes ensuring the following:

* All references, tables, and figures are properly cited

* All relevant supporting information is included in the manuscript submission,

* There are no issues that prevent the paper from being properly typeset

You will receive further instructions from the production team, including instructions on how to review your proof when it is ready. Please keep in mind that we are working through a large volume of accepted articles, so please give us a few days to review your paper and let you know the next and final steps.

Lastly, if your institution or institutions have a press office, please let them know about your upcoming paper now to help maximize its impact. If they'll be preparing press materials, please inform our press team within the next 48 hours. Your manuscript will remain under strict press embargo until 2 pm Eastern Time on the date of publication. For more information, please contact onepress@plos.org.

You will receive an invoice from PLOS for your publication fee after your manuscript has reached the completed accept phase. If you receive an email requesting payment before acceptance or for any other service, this may be a phishing scheme. Learn how to identify phishing emails and protect your accounts at https://explore.plos.org/phishing.

If we can help with anything else, please email us at customercare@plos.org.

Thank you for submitting your work to PLOS ONE and supporting open access.

Kind regards,

PLOS ONE Editorial Office Staff

on behalf of

Dr. I Anna S Olsson

Academic Editor

PLOS One
